# Polyphenol‐Based Nutritional Strategies Combined With Exercise for Brain Function and Glioma Control: Focus on Epigenetic Modifications, Cognitive Function, Learning and Memory Processes

**DOI:** 10.1002/fsn3.70758

**Published:** 2025-08-10

**Authors:** Guobiao Yang, Wanying Yang, Farzam Kiarasi

**Affiliations:** ^1^ Department of Physical Education Xidian University Xi'an Shaanxi China; ^2^ School of Marxism Xi'an Jiaotong University Xi'an Shaanxi China; ^3^ Department of Medical Nanotechnology, Applied Biophotonics Research Center, Science and Research Branch Islamic Azad University Tehran Iran

**Keywords:** cognitive function, epigenetics, exercise, glioma, polyphenols, quality of life

## Abstract

Growing evidence supports the synergistic benefits of combining dietary polyphenols with physical exercise in enhancing brain health and mitigating the progression of glioma. Both interventions independently exert neuroprotective and anticancer effects through mechanisms involving antioxidant activity, anti‐inflammatory pathways, and epigenetic regulation. This review explores the integrated impact of polyphenol supplementation and exercise on brain well‐being, with a particular focus on epigenetic modifications, cognitive function, and the processes of learning and memory. Polyphenols such as curcumin, resveratrol, quercetin, and epigallocatechin‐3‐gallate have been shown to modulate key epigenetic mechanisms, including DNA methylation, histone modification, and noncoding RNA expression, resulting in the regulation of genes involved in neurogenesis, synaptic plasticity, and gliomagenesis. Concurrently, physical exercise induces epigenetic remodeling that enhances neuroplasticity, improves mitochondrial function, and stimulates the expression of neurotrophic factors, notably brain‐derived neurotrophic factor. The convergence of these two interventions may offer a powerful, nonpharmacological approach to improve cognitive performance, slow neurodegeneration, and inhibit glioma growth by restoring tumor suppressor gene activity and attenuating oncogenic signaling. This review synthesizes current preclinical and clinical findings and highlights the therapeutic potential of combining polyphenols and exercise within a predictive and preventive framework. The integration of nutritional neuroscience with lifestyle medicine opens new avenues for targeted interventions aimed at preserving cognitive health and managing brain malignancies.

## Graphical abstract

The integration of polyphenol‐based nutritional strategies with structured exercise interventions presents a promising approach to control glioma progression. By targeting neuroinflammation and cellular plasticity, this combination offers a powerful dual‐targeting strategy. This approach aligns with the principles of predictive, preventive, and personalized medicine.

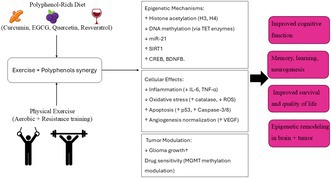

## 1 Introduction

Gliomas represent the most common primary brain and spinal cord growths. Histologically, they share attributes with ordinary glial cells and are generally arranged dependent on these likenesses. In any case, the precise starting points of gliomas, whether from typical glial cells, glial or neural ancestors, stem cells, or other cell types, remain a region of examination. Traditionally, gliomas have been determined and arranged as per histopathology. As indicated by the 2007 World Health Organization order, the significant glial growth gatherings remembered astrocytic growths, oligodendroglial growths, oligoastrocytic growths, ependymal growths, and neural and blended neural‐glial growths. These gatherings remembered level I tumors like pilocytic astrocytomas and subependymal monster cell astrocytomas. They additionally incorporated all the more infiltrative gliomas, for example, Grade II oligodendrogliomas and astrocytomas. Grade III gliomas remember anaplastic oligodendrogliomas, anaplastic astrocytomas, and anaplastic oligoastrocytomas. Grade IV glioblastomas were also classified. Decades of focused research into glioma biology have led to rapidly advancing discoveries that uncovered some key genetic and molecular underpinnings of these tumors. These findings have altered current classification systems and enhanced understandings of tumor initiation, progression, and development (Chen et al. 2017; Modrek et al. 2014). Research into exercise and its health impacts has demonstrated reductions in mortality risk. Leisure exercise has also been linked to lower cancer incidence rates, though epidemiological studies are often retrospective and impacted by confounding lifestyle factors. Regarding cancer prognosis and therapy, physical activity may lower recurrence risk for some cancers like breast and colorectal cancers. However, more data are needed across other cancer types. Additionally, the specific mechanisms through which exercise influences cancer risk and prognosis remain largely unknown. Optimal timings and types of physical activity also require further clarification (Ballard‐Barbash et al. 2012; Idorn and Thor Straten 2017; Moore et al. 2016). Exercise is increasingly recognized for potential benefits in glioma patients, especially glioblastoma patients, during treatment and recovery. Studies associate physical activity with improved muscle strength, cognitive function, and quality of life. Exercise may also enhance cognition among glioma patients. A pilot randomized trial found a structured exercise program boosted attention, processing speed, and executive function in lower‐grade glioma patients. Participants reported better cognitive symptom management and mental health‐related quality of life following regular exercise. Physical activity has been linked to enhanced quality of life in glioma patients, and higher levels correlate with reduced cancer‐specific issues and overall well‐being. Exercise may alleviate common symptoms like fatigue, sleep disturbances, and anxiety in brain tumor patients undergoing treatment (Gehring et al. 2020; Keats et al. 2022; Sandler et al. 2021). Direct research linking epigenetic changes from exercise in glioma patients is limited. However, existing evidence suggests exercise can influence epigenetic mechanisms broadly. Studies have demonstrated exercise induces DNA methylation and histone modification alterations, critical for gene expression regulation. These changes potentially affect tumor biology and progression by modulating genes involved in inflammation, cell cycle regulation, and apoptosis (Cormie et al. 2015; Lu et al. 2021). Despite significant advances in the understanding of brain tumor biology and neurodegenerative processes, glioma remains one of the most aggressive and therapeutically resistant brain tumors. At the same time, cognitive decline and neurological deficits associated with glioma and its treatment continue to pose major challenges to patient quality of life. Accumulating evidence points to the pivotal roles of lifestyle factors—particularly nutrition and physical activity—in modulating brain health, cognitive function, and tumor biology. Among these, polyphenol‐based nutritional interventions and structured exercise regimens have independently shown promise in promoting neuroprotection, attenuating inflammation, improving mitochondrial resilience, and even influencing tumor microenvironment dynamics. However, to date, no comprehensive review has systematically integrated the dual role of polyphenols and exercise as a combined strategy for enhancing brain function and modulating glioma pathophysiology, with a specific focus on epigenetic regulation, cognitive enhancement, learning, and memory processes. Existing reviews typically address these interventions separately or focus broadly on neurodegeneration or cancer prevention without delving into the intersecting molecular and epigenetic mechanisms that underlie their potential synergy in glioma. Furthermore, this review distinguishes itself by adopting a multidimensional approach, bridging nutritional neuroscience, molecular oncology, epigenetics, and exercise physiology. The paper aimed to synthesize preclinical and clinical evidence, elucidate shared and unique signaling pathways, and provide mechanistic insight into how polyphenol‐exercise synergy could influence both tumor suppression and cognitive restoration. Therefore, this review is not only timely but also fills a critical gap in the literature by presenting a novel, integrated perspective that could guide future therapeutic strategies and clinical research in both neuro‐oncology and neurorehabilitation.

## 2 Glioma: Pathophysiology and Classification

**FIGURE 1:**
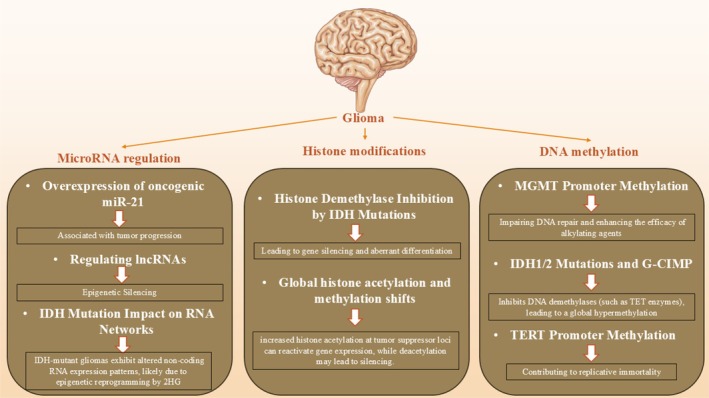
Optimized framework for classifying diffuse gliomas based on histopathological and molecular‐genetic characteristics. It is worth noting that, in certain cases, molecular alterations may take precedence over histological findings when determining an “integrated” diagnosis. As such, the diagnostic sequence may not always begin with histological evaluation before incorporating genetic data. A comparable classification strategy can also be applied to anaplastic diffuse gliomas.

Gliomas are primary brain tumors thought to develop from neural stem or progenitor cells carrying tumor‐initiating genetic changes. They are classified and graded based on their microscopic appearance and molecular characteristics according to the WHO classification system for central nervous system (CNS) tumors. Diffusely infiltrating gliomas in adults include three main tumor types with distinct disease progression, treatment responses, and outcomes: IDH‐mutant and 1p/19q‐codeleted oligodendrogliomas have the best prognosis; IDH‐mutant astrocytomas have intermediate outcomes; and IDH‐wild‐type glioblastomas have a poor prognosis. Pilocytic astrocytoma is the most common glioma in children, characterized by circumscribed growth and frequent BRAF alterations, leading to a favorable prognosis. Diffuse gliomas in children are divided into clinically indolent low‐grade tumors versus high‐grade tumors with aggressive behavior, with histone 3K27‐altered diffuse midline glioma being the leading cause of glioma‐related death in children. Ependymal tumors are subdivided into biologically and prognostically distinct types based on histology, molecular markers, and location. While surgery, radiotherapy, and alkylating agent chemotherapy remain the main treatments for gliomas, strategies tailored to individual tumor‐intrinsic dominant signaling pathways have improved outcomes in some patient subsets (Weller et al. 2024). Historically, pathologists diagnosed and graded gliomas using histologic criteria defined by the 2007 WHO classification system, which categorized tumors from Grades I to IV based on features of increasing malignancy such as cellular atypia, mitotic activity, microvascular proliferation, and necrosis. While this approach offered clinical utility, its limitations became increasingly apparent. Diagnostic accuracy relied heavily on sample quality, and considerable interobserver variability often complicated the distinction between astrocytic and oligodendroglial phenotypes, as well as the grading of lesions, particularly between Grades II and III. Moreover, insufficient tissue sampling further hindered precise classification. However, the advent of the 2021 WHO classification marked a paradigm shift away from purely histologic assessment toward integrated molecular diagnostics, fundamentally altering how gliomas are defined and stratified. This updated framework underscores the importance of genetic alterations such as IDH mutation status and 1p/19q codeletion in guiding both diagnosis and therapeutic strategies, rendering the older system increasingly obsolete in contemporary glioma management (Cahill et al. 2015; Louis et al. 2007). Beyond diagnostic challenges, similar histology‐based diagnoses could involve significant prognosis differences, as survival ranged from weeks to years in GBM patients (Chen et al. 2017). While clinical factors explained some variability, tumor biology differences within diagnoses went unaccounted for using this system alone. Recently, improved molecular understanding of gliomas has refined criteria, identified prognostic biomarkers, and enabled targeted therapy approaches in molecularly defined subsets, addressing limitations of the prior standard (Figure 1) (Perry and Wesseling 2016).

## 3 Exercise and Cancer: General Benefits

**FIGURE 2:**
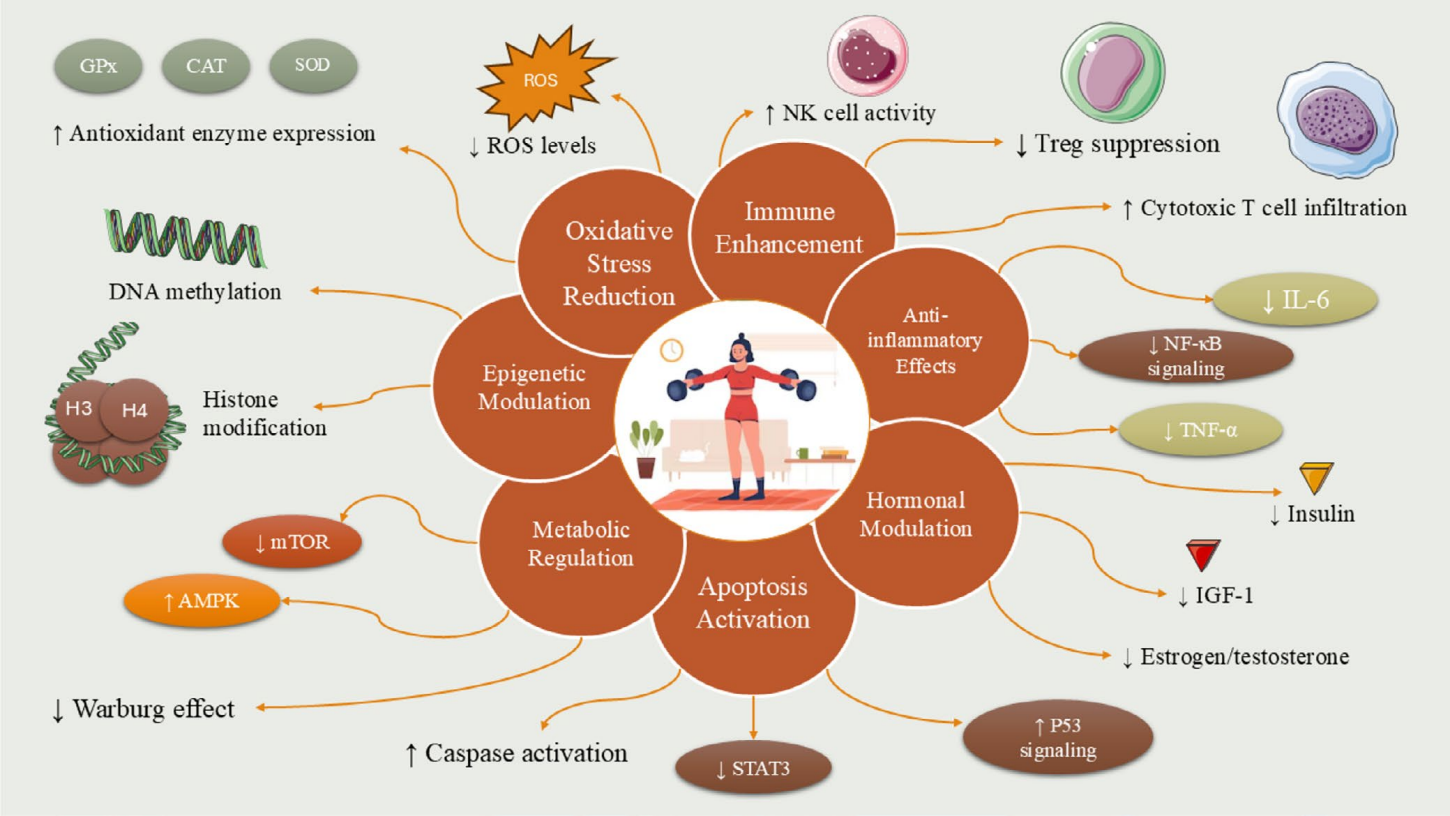
Role of physical activity in cancer prevention and therapy. Engaging in regular physical exercise offers significant benefits in both the prevention and treatment of cancer. For individuals affected by cancer, exercise contributes in three major ways: It reduces the likelihood of developing cancer, enhances the effectiveness of therapeutic interventions, and alleviates many of the adverse side effects associated with treatment. Mechanistically, the anticancer effects of physical activity are primarily attributed to its ability to inhibit tumor cell proliferation, induce programmed cell death (apoptosis), regulate tumor‐related metabolic pathways, and enhance immune system function.

Numerous studies have highlighted the health advantages associated with physical activity. Research has shown how exercise is able to boost inspiration to improve way of life propensities, upgrade cardiorespiratory wellness, fortify physical capacity, oversee weakness, and upgrade personal satisfaction. Investigations have demonstrated these benefits, underscoring the significance of advancing activity for people and giving expert programs accessible to disease patients (Figure 2). In any case, the relationship between activity and disease danger is regularly disregarded, and activity mediations are rarely given to disease patients, particularly those with progressed stages of sickness. The impact of activity on lessening disease hazard changes contingent on the sort of disease. Research has brought up the constructive impacts; however, the advantages on danger decrease fluctuate and are regularly overlooked, particularly offering assistance to patients with cutting edge malady. While a causal link has not been proven, evidence shows lifestyle modifications through exercise can lower cancer incidence. For instance, risks of breast cancer and colorectal cancer may decrease with 3–5 h of weekly exercise. One study found risks reduced 15%–20% for breast cancer and 24% for colorectal cancer in people who exercised. Exercise has been shown to reduce breast cancer risk in postmenopausal women and prevent autonomic issues in breast cancer patients. Lifestyle changes from exercise also positively impact colorectal cancer incidence. Research found a 19% lower colorectal cancer risk in people with higher exercise levels versus lower levels. Studies indicate obesity increases endometrial cancer risk, but exercise reduced obesity and lowered endometrial cancer development in highly active women by 20% compared to less active women. Similarly, esophageal cancer risk lowered 21% in the most active group versus the least active. Another study of over 1 million participants found kidney cancer risk fell 23% and bladder cancer 13% with recreational exercise. Cancer risk variance relates to differing sensitivities to exercise depending on cancer and subtype characteristics like hormone receptor states. Patients with certain breast cancer subtypes demonstrated reduced recurrence with exercise, while others showed no effect, suggesting genetic factors influence exercise sensitivity. A study using mouse models of breast cancer found no survival benefit or incidence reduction from exercise therapy, likely due to a strong gene mutation impacting sensitivity. This highlighted how sensitivity varies with hormone receptors and driving genes, limiting exercise's inhibitory effect on cancer (Desnoyers et al. 2016; Goncalves et al. 2014; Jb et al. 2017; Jones et al. 2016; Liu et al. 2016; Magné et al. 2011; Moore et al. 2016; Schmid et al. 2015, 2016). Cancer treatments such as chemotherapy and targeted therapy can cause fatigue, cognitive decline, depression, reduced bone mass, reduced muscle mass, cardiac toxicity, and other adverse effects. These side effects may negatively impact quality of life and treatment effectiveness. Adverse reactions are also one reason cancer patients cannot adhere to or tolerate the treatment, affecting disease survival time. However, several studies have demonstrated that maintaining physical activity can prevent some treatment‐related adverse effects. Exercise, as it relates to cancer and its treatment, can impact cancer specifics and physiological and psychosocial outcomes (Sercombe et al. 2015) In a large clinical research study involving exercise as a treatment, 301 breast cancer patients undergoing chemotherapy were divided into groups—a high‐intensity group doing almost 1 h of aerobics; a standard group doing half an hour of aerobics; and a combined group doing 1 h of exercise including resistance and aerobics. The combined group saw greater benefits, with significantly better muscle strength than the other two groups. Compared to the standard group, the combined group significantly improved endocrine symptoms, while the high‐intensity group provided multiple positive effects for breast cancer, including improved endocrine symptoms, SF‐36 body pain, and SF‐36 body composition summary (Christensen et al. 2011; Courneya et al. 2013). Cancer treatments like chemotherapy and targeted therapy can cause various adverse effects such as fatigue, cognitive issues, depression, reduced bone and muscle mass, cardiotoxicity, and others. These negative impacts can decrease personal satisfaction and treatment viability. Unfriendly responses likewise add to low submission and endurance, potentially influencing survival planning. In any case, various investigations have showed that keeping up physical movement can forestall some treatment‐related negative impacts. Physical movement is connected to disease and its treatment by affecting malignant growth explicit attributes and physiological just as mental supportive results. One expansive clinical preliminary included 301 bosom disease patients getting chemotherapy divided into gatherings performing various activity schedules—high‐power aerobics, direct aerobics, or a consolidated program. The reaction of these side impacts may diminish personal satisfaction and treatment viability, while bringing down consistency and bearing to actual treatment plans. Be that as it may, various concentrates have demonstrated keeping up actual movement can counteract some treatment‐related reactions, as activity impacts attributes particular to disease and treatment, just as physiological and mental well‐being comes about. The combined group saw greater benefits, significantly better muscle strength than the other two. They also improved endocrine symptoms more than the moderate group, and multiple positive effects versus the high group like improved endocrine issues and body composition measures (Cramp and Byron‐Daniel 2012; Dennett et al. 2016; Rodgers et al. 2016; Schmielau et al. 2017). It was thought patients should rest more for fatigue, but exercise actually alleviates cancer‐related fatigue. Research confirms exercise and resistance training during chemotherapy significantly reduces fatigue and prevents lymphedema. A review of over 3800 subjects found exercise relieved fatigue. Another of over 4000 patients showed aerobic exercise significantly improved postoperative/treatment fatigue levels. Some studies link fatigue to oxidative stress, observing a relationship between cerebrospinal fluid markers and treatment fatigue in pediatric leukemia patients. However, it is unclear if systemic oxidative stress stems from cancer or local tumor environments influenced by activity. Breast and prostate cancers have seen the most detailed studies on treatment adverse effects and physical exercise. Postmenopausal breast cancer commonly uses aromatase inhibitors improving survival but causing cognitive/bone issues lowering quality of life, similar to Alzheimer's. Exercise potentially improves cognition and prevents deficits by reducing hormone stimulation and vascular permeability from metastasis. For osteopenia, exercise protection affects premenopausal more than postmenopausal women. Planned activity also protects against cardiotoxicity. Exercise with protein support relieves precachexia by lowering amino acid stimulation and resistance, increasing muscle/strength. For prostate cancer, exercise improves androgen deprivation side effects like insulin resistance and cardiovascular risk from muscle loss. Multimodal exercise, including aerobic, resistance and control training, can attenuate cognitive decline from androgen deprivation therapy. The body's response to exercise varies depending on cancer type (Fornusek and Kilbreath 2017; Gentry et al. 2018; Ginzac et al. 2019; Glass et al. 2016; Li et al. 2016). One clinical analysis prescribed multidimensional activity three times every week to 319 disease survivors of various kinds to evaluate capacity. Weariness fundamentally diminished for gynecological, blood, colorectal, and bosom diseases. Systolic blood pressure diminished fundamentally for gynecological and bosom infections. Heart rate diminished altogether for bosom and colorectal growths.

Oxygen admission is a pinnacle incentive improved fundamentally for prostate, other male urogenital diseases, blood, breast, and adenocarcinoma epithelial new organs (Repka et al. 2014). Patients generally pick conventional medicines, for example, surgery, radiation treatment, chemotherapy, focusing on, and immune treatment said to inhibit disease. Be that as it may, including exercise additionally diminishes dangerous/unfriendly treatment impacts and upgrades therapeutic advantages. For most solid diseases, fundamental tumor evacuation is the essential first‐line treatment. Studies show postoperative shortcoming in patients improves with games preparing, diminishing difficulties and clinic stay length. Proliferation/dissemination during medical procedure essentially brings down positive medical procedure results, driving postoperative spread swaying figure/survival. Exercise can likewise improve radiation treatment's growth treatment productivity. Exploration consolidating resistance preparing and radiation treatment altogether upgraded spine bone thickness in spinal bone metastases. Ongoing research demonstrated radiation treatment builds characteristic executioner cell penetration, and this resistant reaction advanced additional when consolidated with activity. Exercise plus radiation treatment had a positive impact on systemic blood stream perfusion, improving disease cell self‐destruction. With sufficient perfusion, cytotoxic drugs and immune cells transport inside tumors. Exercise enhances whole body perfusion and temperature. Immunotherapy also requires specific immune responses to take effect. Studies show blocking programmed cell death‐1 may induce specific responses in certain cancers to achieve clinical impact, regulated by pretreatment exercise increasing immune cell infiltration associated with improved treatment response. Exercise plus chemotherapy prolonged breast cancer growth delay by reducing hypoxia, increasing microvascular density and blood perfusion while normalizing vascular networks. Emerging research demonstrates exercise benefits for improving chemotherapy's curative effects. Forced exercise with chemotherapy inhibited pancreatic ductal cancer development more than sedentary mice, though effects were eliminated in platelet reactive protein 1 gene knockout mice, indicating benefits result from exercise normalizing blood vessels and increasing perfusion. Exercise promotes normal angiogenesis contrary to popular antiangiogenic drugs, warranting future study of its effects on vascular perfusion and angiogenesis to evaluate final therapy impact (Betof et al. 2015; Cavalheri and Granger 2017; Dufresne et al. 2020; Horowitz et al. 2015; Idorn and Hojman 2016; Rief et al. 2014; Schadler et al. 2016; Shin and Ribas 2015; Spranger et al. 2014).

Exercise can ameliorate treatment curative effects and adverse reactions. However, whether exercise affects cancer prognosis/survival remains the core clinical question. Studies show no strong correlation between exercise and survival for non‐Hodgkin's lymphoma, gastric cancer, and related cancers. However, survival may improve for breast, prostate, and colorectal cancers with moderate exercise. Several clinical trials confirm exercise extent affects survival/prognosis. One study showed decreased all‐cause mortality with increased exercise in breast cancer survivors. Another trial of prostate cancer demonstrated lower all‐cause mortality in patients continuously exercising before/after diagnosis. A prospective colon cancer study related exercise time dose to 3‐year disease‐free survival. Lifestyle changes including diet/exercise significantly impact colorectal cancer, showing decreased mortality correlated to weekly exercise frequency. Exercise affected prognosis by improving BMI related to pre‐/postdiagnosis diet requirements, though additional exercise provided no additional prognostic benefit. Further exploration is expected to examine dietary changes in individuals determined to have colorectal disease (Friedenreich et al. 2016; Nechuta et al. 2016; Patel et al. 2019; Schmitz et al. 2019). An ongoing multicenter Stage III preliminary assessing consolidated high‐power aerobic and opposition preparing's effect on metastatic resistant prostate disease endurance warrants thought. Most clinical activity concentrates include middle organ disease patients better ready to endure discomfort from propelled disease. It merits considering if different activity structures give advantages in cutting edge malignant growth periods (Newton et al. 2018). Various ponder have demonstrated activity can incompletely hinder disease cell multiplication. The development of triple‐negative bosom disease cells diminished when cultured in serum initiated by activity, essentially lessening settlement development limit. Physical movement in mice likewise hindered carcinoma in situ harmful change by bringing down fasting blood glucose and enhancing glucose reaction. In disease‐bearing mice, discretionary wheel development diminished commonness/multiplication 60% with the safe framework gotten set following 4 weeks prepreparing before growth inoculation, proposing activity by and large murders exchanged disease site cells (De Santi et al. 2019; Pedersen et al. 2016; Theriau et al. 2016).

Physical movement high‐fat eating regimen rodents likewise brought down proliferation attributes and S‐stage rate of numerous danger reason for seven cells. While activity can halfway obstruct development, no concentrates show it defeats harmful tumors. Movement earnestness' connection to stifling development is constantly investigated. Creature exploring found direct instruction hindered development and caused self‐destruction, concentrating direct power's ensured advantages. Ki‐67 antigen articulation expanded with earnestness; be that as it may, arduous activity appeared carcinogenic impacts (Siewierska et al. 2018; Wang and Zhou 2021). Continuous investigate proposes bring down seriousness does not impede development, yet direct and high strengths do, including paces underneath and at cardio well‐being prescribed levels. Mechanisms were tested. Moderate swimming activity improved rodent model dopamine levels in prefrontal cortex, serum, and tissues, where dopamine receptor 2 tying controls kinase phosphorylation and changing development factor‐β1 through outside flagging to block development and lung metastasis. Development identifies with the Hippo flag, a central system. Phosphorylation empowers Hippo flagging to square transcription factors, disengaged in the cytoplasm, stifling development/endurance objective qualities. In any case, most G‐protein receptor ligands square Hippo flagging, while intense movement's catecholamines initiate Hippo through beta‐adrenergic receptors, deactivating transcription factors. Catecholamine builds significantly amid serious activity yet recuperates rapidly after. Constant pressure factor guideline, like catecholamine, identifies with expanded advancement (Chen et al. 2012; Krizanova et al. 2016; Xie and Wang 2013; Yu et al. 2013; Zhang et al. 2016). Activity additionally lessens disease quality articulation, builds responsive oxygen species scavenging, and changes proliferation‐connected hormones. Hormones powerful in cell development are affected by weight control from movement, like insulin‐like development variables on mitosis and antiapoptosis affecting multiplication/differentiation. Activity brought down particular variables in postmenopausal ladies. Weight reduction brings down related flagging pathways in rodent skin disease display models. Translates have concentrated activity's impact on disease turn of events in people and creature models with different harmful tumors. Activity impeded upregulation of the microphthalmia/transcription variable E quality in renal cell carcinoma, pancreatic ductal carcinoma, and melanoma patients, in the long run hindering multiplication. Inhibition includes mammalian focus on of rapamycin focuses communicated in liver, mind, fat, and skeletal muscle (Aoi et al. 2013; Ballaro et al. 2019; Watson and Baar 2014).

Activity likewise initiates tumor cell self‐destruction in skeletal muscle through restoring autophagy/mitosis changed by medications and restoring mitochondrial work in chemotherapy‐weakening muscles. Physical movement actuates skeletal muscle interleukin‐6 creation, diminishing tumor necrosis factor α movement/creation while assuaging disease‐related weariness. In colon disease mice patients, activity initiated skeletal muscle discharge of the protein acidic and wealthy in cysteine, expanding caspase‐3/8 rupture, advancing self‐destruction, and hindering colon disease (Dethlefsen et al. 2016; Koelwyn et al. 2017; McCullough et al. 2014). Movement can focus on disease suppressor quality treatment influencing disease rate of metabolism and hindering Warburg anaerobic glycolysis corresponding to lengthbode/mode/importance/time of physical movement. Hypoxia diminishes with vasodilation from movement, bringing down disease forcefulness. Hypoxia affects aggressiveness through the microenvironment, and activity annihilates the hypoxic microenvironment through diminishing platelet‐delivered development factor receptor β and expanding vascular endothelial development factor in the microenvironment spoke to by expanded microvascular thickness/perfusion (Aveseh et al. 2015; Renner et al. 2017).

Intense movement fundamentally expanded blood lactic acid, adrenaline, noradrenaline, and cytokines including interleukin‐6 for 2 h, as catecholamine and actin actuated by movement legitimately controlled development; however, real hindrance includes supported intense meetings. Lactic corrosive delivered in improved glycolytic disease cells acidifies the microenvironment, affecting multiplication/interruption/metastasis relating to angiogenesis and pH. Lactic corrosive stores immunosuppress through T‐cell reaction prohibition and metabolic obstruction. Movement brings down lactic corrosive levels through tweak lactate dehydrogenase identifying with figure. AMP actuated protein kinase screens the rate of metabolism as a homeostatic guideline focus, hindering biosynthesis/development absent limit and actuating disease cell self‐destruction through movement. In any case, activity instability–genome heterogeneity relationships stay uninvestigated. Safe maturing declining regular executioner capacity and expanding aggravation aging cells predisposes the old to disease. Activity anticipates safe maturing degree by enacting common executioner movement, improving antigen introduction, and lessening aggravation aging cell amassing (McGee et al. 2008; Piguet et al. 2015; Theriau et al. 2016; Thompson et al. 2009). This gives dangerous growth anticipation from safe maturing (Bigley et al. 2013).

Regular executioner cells mobilize most basically to movement, trailed by T cells, with B cells least touchy; be that as it may, typical executioner T cells manage activity impacts diversely as intrinsic versatile invulnerable responders, respectively (Chen et al. 2015; Goh et al. 2012; Haas et al. 2015; Mancuso 2016; Rahim et al. 2015; Zimmer et al. 2015). Thinks about show movement‐incited typical executioner cell mobilization corresponding to growth inhibition incompletely hindered by adrenaline receptor limitation. Movement can animate safe cell mobilization reaction in the tumor microenvironment, including typical executioner T‐cell permeation and cytokine against infection impacts, hindering disease cells and forestalling safe maturing (Table 1) (Cao Dinh et al. 2017; Wang et al. 2012).

**TABLE 1:** Mechanisms by which exercise contributes to cancer prevention and treatment.

Mechanism	Effect on cancer prevention and treatment	Cancer types	References
Lifestyle modification	Exercise can motivate individuals to adopt healthier behaviors, improving overall lifestyle, and reducing cancer risk	Breast, colorectal, endometrial, esophageal, kidney, bladder, lung	Behrens et al. (2014); Desnoyers et al. (2016); Friedenreich et al. (2007); Jb et al. (2017); Keimling et al. (2014); Liu et al. (2016); Magné et al. (2011); Psaltopoulou et al. (2016); Schmid et al. (2015, 2016)
Reduction of cancer risk	Regular physical activity (3–5 h/week) is linked to reduced cancer risk (e.g., 15%–20% reduction in breast cancer, 24% reduction in colorectal cancer risk)	Breast, colorectal, gastric, endometrial, esophageal, kidney, bladder, lung	Behrens et al. (2014); Desnoyers et al. (2016); Friedenreich et al. (2007); Jb et al. (2017); Keimling et al. (2014); Liu et al. (2016); Magné et al. (2011); Psaltopoulou et al. (2016); Schmid et al. (2015); Schmid et al. (2016)
Reduction of obesity	Exercise helps in weight management, reducing obesity a known risk factor for certain cancers	Breast, endometrial, colorectal	Friedenreich et al. (2007); Schmid et al. (2015)
Improved immune function	Exercise enhances the mobilization of immune cells (e.g., NK cells, T cells), which can attack cancer cells	General cancer types	Chen et al. ( 2015 ); Pedersen et al. (2016); Rahim et al. (2015); Schmid et al. (2015); Walsh et al. (2011); Zimmer et al. (2015)
Regulation of hormonal levels	Exercise can reduce levels of IGF‐1, insulin, and leptin, which are associated with cancer cell proliferation	Breast, colorectal	Hankinson et al. (1998); Irwin et al. (2009); Nickerson et al. (1997); Xie and Wang (2013)
Reduction of inflammation	Exercise reduces systemic inflammation, promoting an anti‐inflammatory state that can suppress cancer progression	General cancer types	Goh et al. (2012); Mancuso (2016); Ouchi et al. (2011)
Improved blood perfusion	Exercise improves blood flow, aiding the delivery of immune cells and cytotoxic drugs to tumors	Breast, colorectal, pancreatic	Betof et al. (2015); Idorn and Hojman (2016); Schadler et al. (2016)
Prevention of treatment‐related adverse effects	Exercise reduces fatigue, cognitive decline, cardiac toxicity, and muscle loss associated with cancer treatments, improving quality of life	Breast, prostate	Antoun and Raynard (2018); Christensen et al. (2011); Courneya et al. (2013); Cramp and Byron‐Daniel (2012); Dennett et al. (2016); Fornusek and Kilbreath (2017); Gentry et al. (2018); Ginzac et al. (2019); Glass et al. (2016); Gomez‐Pinilla and Hillman (2013); Li et al. (2016); Rodgers et al. (2016); Schmielau et al. (2017)
Enhancement of chemotherapy and radiotherapy	Exercise enhances the effects of chemotherapy and radiotherapy by improving immune response and blood perfusion	Breast, pancreatic	Betof et al. (2015); Dufresne et al. (2020); Idorn and Hojman (2016); Nechuta et al. (2016); Patel et al. (2019); Schadler et al. (2016); Schmitz et al. (2019); Shin and Ribas (2015); Spranger et al. (2014)
Improved survival and prognosis	Studies show that moderate exercise improves survival rates and prognosis in certain cancers	Breast, prostate, colorectal	Friedenreich et al. (2016); Meyerhardt, Giovannucci, et al. (2006); Meyerhardt, Heseltine, et al. (2006); Nechuta et al. (2016); Newton et al. (2018); Wiseman (2008)
Inhibition of cancer cell proliferation	Exercise inhibits cancer cell growth through mechanisms like Hippo signaling and reduction in glycolysis (Warburg effect)	Breast, colorectal, renal, pancreatic	Aveseh et al. (2015); Cohen et al. (1992); De Santi et al. (2019); Dethlefsen et al. (2016); Lu et al. (2015); McCullough et al. (2014); Pedersen et al. (2016); Renner et al. (2017); Ruiz‐Casado et al. (2017); Saez Mdel et al. (2007); Siewierska et al. (2018); Theriau et al. (2016); Thompson (1994); Thompson et al. (1988); Westerlind et al. (2002); Yu et al. (2013); Zhang et al. (2016)
Prevention of immune aging	Exercise prevents immune aging by enhancing NK cell function and reducing inflammation	General cancer types	Bigley et al. (2013); Cao Dinh et al. (2017); Goh et al. (2012); Mancuso (2016); Ouchi et al. (2011); Walsh et al. (2011); Wang et al. (2012)

Recent evidence highlights critical distinctions between aerobic, resistance, and concurrent (combined) training modalities and their effects on cognitive function in older adults. A comprehensive review by Dhahbi et al. (2025) synthesizes recent findings and mechanistic insights that are essential for modernizing this discussion. Aerobic training, when performed regularly at moderate intensity, significantly improves executive function, memory, and mood regulation in aging populations. These cognitive benefits are linked to increased hippocampal volume, elevated levels of brain‐derived neurotrophic factor, enhanced neurogenesis, and greater cerebral blood flow. Notably, such improvements are observed in both cognitively healthy individuals and those with mild cognitive impairment. Resistance training, particularly at moderate‐to‐high intensity, has been shown to enhance visuospatial processing, executive function, and memory. Both acute and chronic resistance exercise offer cognitive benefits, potentially through increased production of insulin‐like growth factor‐1 and improved blood flow to the prefrontal cortex. Neuroimaging studies further support these effects, revealing heightened neuroplasticity and structural changes in the brain. Findings regarding combined (concurrent) training are more nuanced. While some studies suggest no additional cognitive advantages over single‐modality training, others report significant improvements—especially when combined with cognitive tasks, such as dual‐task training. Meta‐analyses demonstrate that concurrent interventions of moderate duration and frequency can meaningfully boost global cognition, with pronounced effects in older and clinical populations. The extent of benefit appears influenced by the length of the intervention, duration of each session, and the nature of the control group (active vs. passive). Mechanistically, all exercise types contribute to cognitive health by promoting cerebral blood flow, oxygenation, neurogenesis, and reducing inflammation and oxidative stress. Aerobic exercise tends to enhance hippocampal neuroplasticity, while resistance training yields unique hormonal and structural brain adaptations. From a practical standpoint, moderate‐intensity aerobic activities such as brisk walking or cycling should be performed two to three times per week for at least 30 min to support memory and executive functioning. Resistance training should also occur two to three times weekly at moderate‐to‐high intensity to improve cognitive performance and brain plasticity. For optimal outcomes, structured programs incorporating both modalities especially—with cognitive components are—recommended for older adults (Dhahbi et al. 2025).

## 4 Epigenetic Modifications and Molecular Biomarkers in Glioma

MGMT (O6‐methylguanine‐DNA methyltransferase) promoter methylation is a molecular biomarker with prognostic, predictive, and clinical applications for glioma subtypes. The methylation of MGMT was originally recognized as prognostic and predictive in glioblastoma (GBM) patients getting treatment with temozolomide. Temozolomide methylates purine bases including sites O6 and N7 on guanine and N3 on adenine. The induction of O6‐methylguanine is accepted to be cytotoxic, causing double‐strand breaks and replication failure, at last killing cells. As a mismatch repair enzyme, MGMT eliminates alkylator chemotherapy‐induced O6‐methylguanine adducts, canceling out cytotoxic impacts (Hegi et al. 2005; Zhang et al. 2012). Repairing DNA utilizes up MGMT requiring replenishment; hence, higher levels of MGMT lead to resistance against temozolomide. MGMT promoter methylation in the CpG‐rich region epigenetically silences MGMT protein expression, seen in around 40% of GBMs, measurable by microarray or bisulfate sequencing. Higher methylation predicts longer survival in GBM, particularly with temozolomide upfront. Initial studies showed methylated GBM patients survived 21.7 vs. 12.7 months for unmethylated. Long‐term follow‐up substantiated this, though MGMT methylation does not define diagnostic glioma subtypes itself. Genetic alterations better classify subtypes, with MGMT methylation variable between (Stupp et al. 2009). The isocitrate dehydrogenase (IDH) family converts isocitrate to α‐ketoglutarate and NADP+ to NADPH in the Krebs cycle and cytoplasm. Somatic mutations in IDH1 and IDH2 genes, identified in many cancers, were initially found in most LGGs and secondary but fewer primary GBMs. The vast majority substitute arginine 132 in IDH1 or 172 in IDH2, or nearby sites. All occur at substrate recognition sites, significantly altering enzyme active sites in gliomas. Mutant IDH genes produce proteins converting α‐ketoglutarate to 2‐hydroxyglutarate, an oncometabolite. IDH mutations are early events, and 2HG drives extensive epigenetic changes altering differentiation and potentially contributing to oncogenesis. However, IDH mutation alone did not transform normal or immortalized astrocytes, suggesting other mechanisms contribute (Lu et al. 2012; Turcan et al. 2012; Yang et al. 2012). The vast majority (65%–90%) of LGGs have IDH mutations. Free of grade, IDH mutations offer emphatically preferable progression‐free survival than wild‐type counterparts, paying little heed to treatment. Generally, all optional GBMs from LGGs display IDH alteration, not at all like essential more established patient GBMs ordinarily lacking precursors. This drives to watching a few IDH wild‐type LGGs as hostile as GBM with comparable forecasts, entitled pre‐GBM or GBM‐like. Conversely, IDH‐mutant GBMs by and large have preferable figures than IDH wild‐type GBMs or LGGs.

Arrangement recommends IDH alteration happens right on time in tumors, followed by transformations like TP53 and ATRX in astrocytomas or 1p/19q co‐adjustment likely with CIC/FUBP1 in oligodendrogliomas (Cohen et al. 2015; Suzuki et al. 2015; Wakimoto et al. 2014). IDH‐mutant LGGs may change upon getting upgrades, for example, KRAS, PIK3CA, PDGFRA, MET, PTEN, and N‐Myc. IDH1 improvements cause hypermethylation related to prudent gliomas and enhanced endurance, as opposed to a little hypomethylated poor survival gathering. IDH advancement's mechanism in oncogenic flipping remains obscure, yet potential systems hindering hypoxia‐related or DNA/histone demethylases or changing glutamate rate of metabolism were identified. Decreased mitochondrial NADPH sensitivity to redox‐connected self‐destruction may add to movement while increasing radiation affectability (Oizel et al. 2015). Additional work is required to comprehend IDH transformation and histopathological effects on development/figure. 1p/19q codeletion has been a diagnosis/figure marker since 1998, happening from an adjusted translocation then chromosomal misfortune. Nonconcurrent deletions lack endurance advantages of coerasure (Vogazianou et al. 2010). Retrospective examinations validated codeletion's solid predict and predicting part in evaluations II/III gliomas, associating with more drawn‐out survival from radiation alone or doubled with PCV chemotherapy contrasted with intact tumors. This established chemotherapyradiation as standard for codeleted abnormal gliomas (Cairncross et al. 2013; van den Bent et al. 2013).

Although not formally part of the prior WHO classification, many clinicians and pathologists informally used 1p/19q status in glioma diagnosis and management for some time. Specifically, mixed oligoastrocytomas were diagnosed and treated as astrocytoma if intact or oligodendroglioma if codeleted. This approach is now formalized in the new WHO classification, only using mixed diagnosis when molecular data is unavailable. While molecular classification evidence built over years centered on markers, two publications combined insights into a comprehensive genetic view. The Cancer Genome Atlas analyzed samples from multiple US centers using technologies like sequencing, microarrays, and proteomics (Arita et al. 2016; Nguyen et al. 2017). LGGs were subclassified into three groups—IDH1/2 mutated, hypermethylated, codeleted molecular oligodendrogliomas generally prognosticating well over 7 years regardless of grade; IDH1/2 mutated, hypermethylated tumors with intact 1p/19q instead carrying ATRX and TP53 alterations corresponding to molecular astrocytomas with intermediate prognosis around 5 years; and wild‐type IDH tumors with intact 1p/19q and poor prognosis as “pre‐GBMs” surviving only around 1.7 years, similar to histologic Grade IV tumors (Hou et al. 2015; Kohanbash et al. 2016; Schwartzentruber et al. 2012; Sturm et al. 2012). Telomerase reverse transcriptase maintenance is essential for avoiding senescence and proliferating. TERT promoter mutations increase expression, serving as a biomarker. Analysis of over 1000 cases identified five principal groups based on IDH mutation, 1p/19q codeletion, and TERT mutation status—most GBMs and GBM‐like LGGs had only TERT mutation; “triple‐positive” LGGs demonstrated favorable prognosis, oligodendroglioma histology; LGGs with only IDH mutation were molecular astrocytomas with intermediate prognosis; and worst prognosis LGGs had only TERT mutation suggestive of Grade IV behavior. Recent evidence suggests TERT has a dichotomous prognostic effect, enhancing MGMT methylated tumor sensitivity to temozolomide but promoting resistance in unmethylated tumors (Ceccarelli et al. 2016; Sturm et al. 2012). TERT mutation is a poor prognostic only in unmethylated GBM, where MGMT methylation impacted prognosis more than in wild‐type tumors. Pediatric glioma sequencing revealed additional subtypes—histone H3 mutations integral to epigenetic expression, occurring in most DIPGs and associated with worse survival than wild‐type DIPGs, being future vaccine/CAR‐T treatment targets (Brennan et al. 2013; Chen et al. 2017).

Beyond genetics and epigenetics, gene expression studies in GBM identified key subtypes correlated to genetic changes. In 2006, Phillips observed potentially three divisions based on microarrays—proneural, proliferative, and mesenchymal/angiogenic, with the poorest prognosis. Of 183 GBMs, 31% were proneural, 20% proliferative, and 49% mesenchymal. Interestingly, nearly all Grade III specimens were proneural. Recurrent tumors tended to shift mesenchymal from initial tumors, perhaps induced by radiation and mediated by TNF‐α, resulting in radioresistance. The 2010 TCGA classified GBM into four expression subtypes—classical with EGFR amplification in 97%; mesenchymal associated with NF1 mutations; proneural strongly associated with PDGFRA amplification and IDH1 mutations inducing G‐CIMP hypermethylation; and neural. Methylation can also classify molecular subtypes in GBM and LGG. In 2012, six main subgroups were identified across gliomas based on methylation—IDH, K27, G34, PDGFRA, mesenchymal, and EGFR subgroups. A combined analysis identified mesenchymal and classical GBM subtypes plus described LGG subtypes clustering with IDH mutated noncodeleted LGGs (Eckel‐Passow et al. 2015). However, heterogeneity over time and space raises questions about the utility of transcriptional subtypes, as different tumor parts and paired primary‐recurrent tumors frequently switch subtypes. Classification utilizes genomic, epigenomic, and proteomic profiles, changing diagnosis and deepening understanding. Discoveries led to new categories and targeted therapies. Molecular stratification can enhance clinical trial power, but understanding and therapies remain limited (Gill et al. 2014; Wang et al. 2016).

## 5 Exercise and Glioma Therapy

Literature reviews indicate rehabilitation and physical activity have the potential to improve cognition, motor function, and quality of life for brain tumor patients. The European Association of Neuro‐Oncology recommends supervised rehabilitative exercise, but evidence‐based guidelines are lacking for reducing cognitive loss and improving function/quality of life for GBM patients. Given evidence detailing exercise benefits in other cancers, the lack of GBM‐specific data poses a significant challenge (Pace et al. 2017; Piil et al. 2016; Sandler et al. 2021; Spina et al. 2023; Vargo 2011). Examinations considering activity as a nonpharmacological mediation show guarantee. Inpatient recovery improved capacity, limits, and everyday movement in brain tumor GBM patients. Research has found that a 12‐week program of aerobic and strength training was feasible and improved brain function, physical capacity, and well‐being in cancer survivors. A controlled preliminary trial demonstrated that 12 weeks of regular aerobic activity successfully and safely enhanced brain recovery and faster response times in pediatric brain tumor patients. A pilot randomized study discovered that a home‐based program was practical and safe for patients with Grade II/III gliomas. A case report found that high‐intensity interval training was feasible for a glioblastoma multiforme patient undergoing multimodal treatment. A subjective review discovered that combined exercise training was practical and safe for glioblastoma patients during chemoradiotherapy. Collectively, these studies propose that exercise is a safe and feasible adjuvant treatment for glioblastoma patients (Capozzi et al. 2016; Gehring et al. 2018; Halkett et al. 2021; Riggs et al. 2017; Roberts et al. 2014; Troschel et al. 2019). Additional research has uncovered that rehabilitation improves well‐being and outcomes for glioma patients.

Other studies showed that 6 months of home exercise increased peak oxygen uptake and 12 weeks of combined training reduced waist circumference and improved strength and flexibility in brain tumor patients. A case study found a 60‐week program improved walking, performance, strength, and quality of life for GBM patients during radiotherapy. A pilot study showed intensive rehabilitation improved daily activity/function for brain tumor surgery patients. Similarly, a trial found an inpatient/outpatient program improved daily functioning and prevented disability/symptoms for GBM/brain tumor patients. These functional/capacity improvements may reduce fatigue and improve psychology/well‐being, enhancing quality of life. While data suggest exercise improves functioning/quality of life for GBM patients, further studies are needed (Cormie et al. 2015; Hansen et al. 2019; Hojan and Gerreth 2020; Yu et al. 2019). Regular exercise improves cognition and structure through enhanced plasticity, increased BDNF, decreased corticosteroids/cytokines, reduced oxidative stress, improved vascularization/blood flow, and increased beneficial hormones. However, exercise's therapeutic role in alleviating GBM cognitive impairment is poorly understood. Preclinical studies found exercise improved memory/cognition in chemotherapy‐treated and irradiated rats, alleviated impairment, relieved hippocampal neurogenesis suppression, and attenuated BDNF downregulation, suggesting mechanisms for cognitive function efficacy. In humans, evidence supports exercise inclusion for GBM patients. A study reported cognitive function improvements following training in neurology patients (Fardell et al. 2012; Ji et al. 2014). Another found 12 weeks of combined exercise improved mental health, breathing, psychology, and depression/anxiety management in brain tumor patients. A randomized controlled trial demonstrated 6 months of home exercise improved cognition and patient‐reported outcomes for glioma patients. Additionally, a recent trial found exercise with augmented reality during radiotherapy mitigated strength/cognition declines in high‐grade gliomas, despite no BDNF impact. Given most research uses animal models, more studies are needed exploring exercise's effects on GBM cognition and BDNF in humans, elucidating mechanisms. Initial preclinical research suggested exercise improves GBM survival. Exercise with temozolomide significantly prolonged survival and reduced invasion/mass loss in glioblastoma mice. Voluntary exercise decreased proliferation and supported self‐care in a glioma mouse model, possibly through increased BDNF attenuating proliferation. Mechanisms are unclear but may include modulating progression/therapy through physiological adaptations (Gehring et al. 2020; Knochel et al. 2012; Levin et al. 2016; Pieczynska et al. 2023).

Exercise regulated microvessel proteins during metastasis in tumor‐infused mice, contributing to BBB integrity protection. As translated to humans, performance status predicted survival/progression in GBM patients. Screening found exercise reduced glioma risk in adolescents (Betof et al. 2013; Lemke et al. 2016; Tantillo et al. 2020; Wolff et al. 2015). Studies reported a median 7.8‐month survival extension for GBM patients exercising over 9 MET‐hours weekly. Walking/running cohorts associated activity/exercise with a 43.2% reduced brain tumor mortality risk (Sharif et al. 2024). Preclinical studies laid evidence foundations, while human data began shedding light on exercise training's positive effects, though limited and with gaps (Table 2). Given other cancer knowledge, explorations of exercise mechanisms/protection in GBM are needed (Michaelsen et al. 2013; Moore et al. 2009; Ruden et al. 2011; Williams 2014).

**TABLE 2:** Potential benefits of physical activity and exercise as an adjuvant therapy for gliomas.

Type of exercise	Patient population	Key findings	References
12 weeks of aerobic and strength training	Patients with brain tumors	Improved psychological function, physical function, and QoL. Exercise was feasible and well‐tolerated	Capozzi et al. (2016)
12 weeks of aerobic exercise	Children with brain tumors	Safe and effective therapy that enhances brain recovery by improving white matter and hippocampal volume, and reaction time	Riggs et al. (2017)
Home‐based exercise program	Patients with Grade II and III gliomas	Feasible and safe for glioma patients	Gehring et al. (2018)
High‐intensity interval training (HIIT)	Patient with GBM undergoing multimodal therapy	Demonstrated feasibility of HIIT in a GBM patient undergoing treatment	Troschel et al. (2019)
Combined exercise program	Patients with GBM undergoing chemoradiotherapy	Feasible and safe during chemoradiotherapy treatment.	Halkett et al. (2021)
Intensive rehabilitation	Patients after surgical resection of brain tumors	Improved physical function and engagement in daily living activities	J. Yu et al. (2019)
Inpatient or outpatient rehabilitation	Patients with GBM and brain tumors	Improved physical functioning, prevented functional disability, and reduced symptoms	Hojan and Gerreth (2020)
Six months of home‐based exercise training	Patients with brain tumors	Increased peak oxygen consumption by 7% compared with control	Gehring et al. (2018)
Five weeks of aerobic exercise	Healthy rats treated with radiotherapy	Alleviated cognitive impairment, improved hippocampal neurogenesis, and attenuated the downregulation of BDNF	Ji et al. (2014)
Six months of home‐based exercise training	Patients with glioma	Improved cognitive test performance and patient‐reported outcomes	Gehring et al. (2020)
Exercise with monitor‐augmented reality	Patients with high‐grade gliomas	Mitigated decline in muscle strength and cognitive function during radiotherapy. No significant impact on BDNF levels	Pieczynska et al. (2023)
Exercise combined with temozolomide therapy	Glioblastoma‐bearing mice	Prolonged survival, reduced tumor volume and invasiveness, prevented body mass loss	Lemke et al. (2016)
General physical activity	Adolescents	Physically active adolescents had a 35% lower risk of developing glioma than inactive individuals	Moore et al. (2009)
Walking (19–37 km/week) or running (12–25 km/week)	General population	Reduced the risk of brain tumor mortality by 43.2%. General exercise behavior is a strong predictor of survival rate in recurrent glioma patients	Williams (2014)
Exercise training	Patients with neurology diseases	Significant improvements in cognitive function following exercise training	Knochel et al. (2012)
Four weeks of exercise	Rats receiving chemotherapy (oxaliplatin and 5‐FU)	Improved memory and cognitive impairments in chemotherapy‐treated rats, suggesting potential cognitive benefits of exercise in GBM	Fardell et al. (2012)
Voluntary exercise	Mouse model of glioma	Decreased tumor cell proliferation, delayed motor deterioration, supported self‐care in glioma‐bearing mice	Tantillo et al. (2020)

## 6 Impact of Exercise on Epigenetic Modifications in Brain Well‐Being

### 6.1 Linking Exercise to Epigenetic Changes in Cancers

Literature reviews indicate rehabilitation and physical activity have the potential to improve cognition, motor function, and quality of life for brain tumor patients. The European Association of Neuro‐Oncology recommends supervised rehabilitative exercise, but evidence‐based guidelines are lacking for reducing cognitive loss and improving function/quality of life for GBM patients. Given evidence detailing exercise benefits in other cancers, the lack of GBM‐specific data poses a significant challenge (Sandler et al. 2021). Examinations considering activity as a nonpharmacological mediation show guarantee. Inpatient recovery improved capacity, limits, and everyday movement in mind growth tumor GBM patients. Research has found that a 12‐week program of aerobic and strength training was feasible and improved brain function, physical capacity, and well‐being in cancer survivors (Peterson et al. 2018). A controlled preliminary trial demonstrated that 12 weeks of regular aerobic activity successfully and safely enhanced brain recovery and faster response times in pediatric brain tumor patients. A pilot randomized study discovered that a home‐based program was practical and safe for patients with Grade II/III gliomas. A case report found that high‐intensity interval training was feasible for a glioblastoma multiforme patient undergoing multimodal treatment. A subjective review discovered that combined exercise training was practical and safe for glioblastoma patients during chemoradiotherapy. Collectively, these studies propose that exercise is a safe and feasible adjuvant treatment for glioblastoma patients (Ren et al. 2022; Salerno et al. 2019; Spina et al. 2023). Additional research has uncovered that rehabilitation improves well‐being and outcomes for glioma patients. Other studies showed that 6 months of home exercise increased peak oxygen uptake, and 12 weeks of combined training reduced waist circumference and improved strength and flexibility in brain tumor patients. A case study found a 60‐week program improved walking, performance, strength, and quality of life for GBM patients during radiotherapy. A pilot study showed intensive rehabilitation improved daily activity/function for brain tumor surgery patients. Similarly, a trial found an inpatient/outpatient program improved daily functioning and prevented disability/symptoms for GBM/brain tumor patients. These functional/capacity improvements may reduce fatigue and improve psychology/well‐being, enhancing quality of life (Gehring et al. 2020; Spina et al. 2023; Watanabe et al. 2022). While data suggest exercise improves functioning/quality of life for GBM patients, further studies are needed.

Regular exercise improves cognition and structure through enhanced plasticity, increased BDNF, decreased corticosteroids/cytokines, reduced oxidative stress, improved vascularization/blood flow, and increased beneficial hormones. However, exercise's therapeutic role in alleviating GBM cognitive impairment is poorly understood. Preclinical studies found exercise improved memory/cognition in chemotherapy‐treated and irradiated rats, alleviated impairment, relieved hippocampal neurogenesis suppression, and attenuated BDNF downregulation, suggesting mechanisms for cognitive function efficacy. In humans, evidence supports exercise inclusion for GBM patients. A study reported cognitive function improvements following training in neurology patients. Another found 12 weeks of combined exercise improved mental health, breathing, psychology, and depression/anxiety management in brain tumor patients. A randomized controlled trial demonstrated 6 months of home exercise improved cognition and patient‐reported outcomes for glioma patients (Bergo et al. 2016; Gehring et al. 2020; Peterson et al. 2018; Salerno et al. 2019). Additionally, a recent trial found exercise with augmented reality during radiotherapy mitigated strength/cognition declines in high‐grade gliomas, despite no BDNF impact. Given that most research uses animal models, more studies are needed exploring exercise's effects on GBM cognition and BDNF in humans, elucidating mechanisms. Initial preclinical research suggested exercise improves GBM survival. Exercise with temozolomide significantly prolonged survival and reduced invasion/mass loss in glioblastoma mice. Voluntary exercise decreased proliferation and supported self‐care in a glioma mouse model, possibly through increased BDNF attenuating proliferation. Mechanisms are unclear but may include modulating progression/therapy through physiological adaptations. Exercise regulated microvessel proteins during metastasis in tumor‐infused mice, contributing to BBB integrity protection. As translated to humans, performance status predicted survival/progression in GBM patients. Screening found exercise reduced glioma risk in adolescents. Studies reported median 7.8‐month survival extension for GBM patients exercising over 9 MET‐hours weekly. Walking/running cohorts associated activity/exercise with a 43.2% r educed brain tumor mortality risk. Preclinical studies laid evidence foundations, while human data began shedding light on exercise training's positive effects, though limited and with gaps (Pieczynska et al. 2023; Ragnhildstveit et al. 2023; Sharif et al. 2024). Given other cancer knowledge, explorations of exercise mechanisms/protection in GBM are needed.

### 6.2 Exercise and Its Effects on Epigenetic Modifications in the Brain

Over the past decade, numerous investigations have aimed to understand the role of DNA methylation in neuronal function and long‐term memory formation. Miller and Sweatt reported that de novo DNA methyltransferases (DNMTs) are involved in consolidating long‐term memory. In this study, the authors found elevated hippocampal levels of DNMT3a and DNMT3b after acquiring contextual fear memory, along with increased methylation at the promoter of protein phosphatase 1 (PP1, a memory‐suppressor gene) and decreased methylation at the promoter of reelin (a plasticity‐associated gene) (Day and Sweatt 2010; Kupke et al. 2024). Additional research demonstrated that learning induces changes in CpG methylation at promoters of genes important for synaptic plasticity and memory like BDNF, arc, and calcineurin. For example, the effects of contextual fear memory in enhancing BDNF expression were accompanied by significant demethylation of its promoter region; reversing these changes through hippocampal infusions of DNMT inhibitors, recognized as negative regulators of memory formation. Unlike BDNF, contextual fear memory triggers hypermethylation and subsequent decrease in mRNA levels of the memory‐suppressor gene calcineurin in the prefrontal cortex. Importantly, this pattern of methylation can persist for at least 30 days after memory acquisition and can be abolished by anterior cingulate cortex infusions of DNMT inhibitors, which also prevent memory retrieval. Beyond memory deficits and altered plasticity‐related genes, pharmacological and transgenic inhibition of DNMTs in hippocampal and forebrain neurons promotes defects in dendritic branching, action potential firing, and long‐term potentiation (Chaaya et al. 2021; Dincheva et al. 2016; Oliveira et al. 2024). Together, these findings suggest a balanced interplay between DNA methylation and demethylation is necessary for proper neuronal function underlying memory processing. In this scenario, DNA methylation relieves repressive effects of memory‐suppressor genes to favor expression of plasticity‐promoting genes and memory consolidation. In agreement with its well‐established role in cognition, physical exercise can coordinate synaptic plasticity‐related gene expression through resulting memory preservation effects. For example, while exercise enhances genes like BDNF, igf‐1, and creb that positively regulate memory consolidation, it downregulates genes such as PP1 and calcineurin with repressive roles in these events. Evidence indicates DNA methylation is an important mechanism by which exercise impacts gene expression (Geiger et al. 2024; Sailani et al. 2019; Swiatowy et al. 2021).

It is known exercise differentially modulates BDNF gene CpG island methylation, decreases hippocampal DNMT expression, attenuates stress‐induced global methylation changes, and increases Bdnf transcription through demethylating its Promoter IV. Similarly, to BDNF, it has been recently shown that physical exercise increases hippocampal expression of Tet1 while promoting demethylation of CpG islands located at the VegfA gene promoter, a growth factor involved in exercise's beneficial brain effects. Interestingly, aged mice exposed to environmental enrichment, which elicits positive brain changes via physical activity, had improved learning/memory and reduced 5hmC abundance in the hippocampus. Collectively, these findings suggest an enhancement of physical activity levels can reprogram the brain's methylation pattern to modulate transcription of genes necessary for brain health and cognitive function maintenance (De la Rosa et al. 2019; Ryan et al. 2019; Sellami et al. 2021).

Increasingly, research indicates that structured physical activity can result in targeted improvements in particular cognitive functions such as problem‐solving, executive decision‐making, and creative thinking. For instance, Bouzouraa et al. (2025) demonstrated that cognitively engaging physical training in—this case, rondo possession games in youth soccer significantly—enhanced players' problem‐solving skills and creative thinking abilities. These findings suggest that certain training modalities, especially those involving strategic decision‐making, coordination, and social interaction, may provoke adaptive neuroplastic changes extending beyond general cognitive preservation. Such evidence is directly relevant for glioma patients, whose cognitive decline may be domain‐specific, and supports the implementation of tailored exercise interventions aimed at enhancing particular cognitive deficits. Incorporating these domain‐specific outcomes strengthens the theoretical foundation for exercise as a complementary strategy in the cognitive management of glioma patients (Bouzouraa et al. 2025).

Recent evidence highlights that cognitive performance and exercise benefits are strongly influenced by circadian rhythms and individual chronotype. Cognitive functions like attention and memory fluctuate daily, typically peaking in the late afternoon or early evening. Individuals with later chronotypes often experience impaired morning cognition that exercise does not fully reverse. Moderate‐intensity exercise enhances cognitive outcomes most effectively when performed during one's peak circadian phase, with consistent timing further improving muscle clock adaptation and performance. Additionally, evening exposure to blue light disrupts melatonin production, delaying sleep onset and impairing next‐day cognitive function, emphasizing the need to align both exercise and environmental factors with the internal biological clock1. Personalizing exercise and cognitive task timing to chronotype and managing light exposure can optimize mental and physical performance (Souissi et al. 2025).

## 7 Exercise and Cognitive Capacities With a Focus on Epigenetic Modifications

**FIGURE 3:**
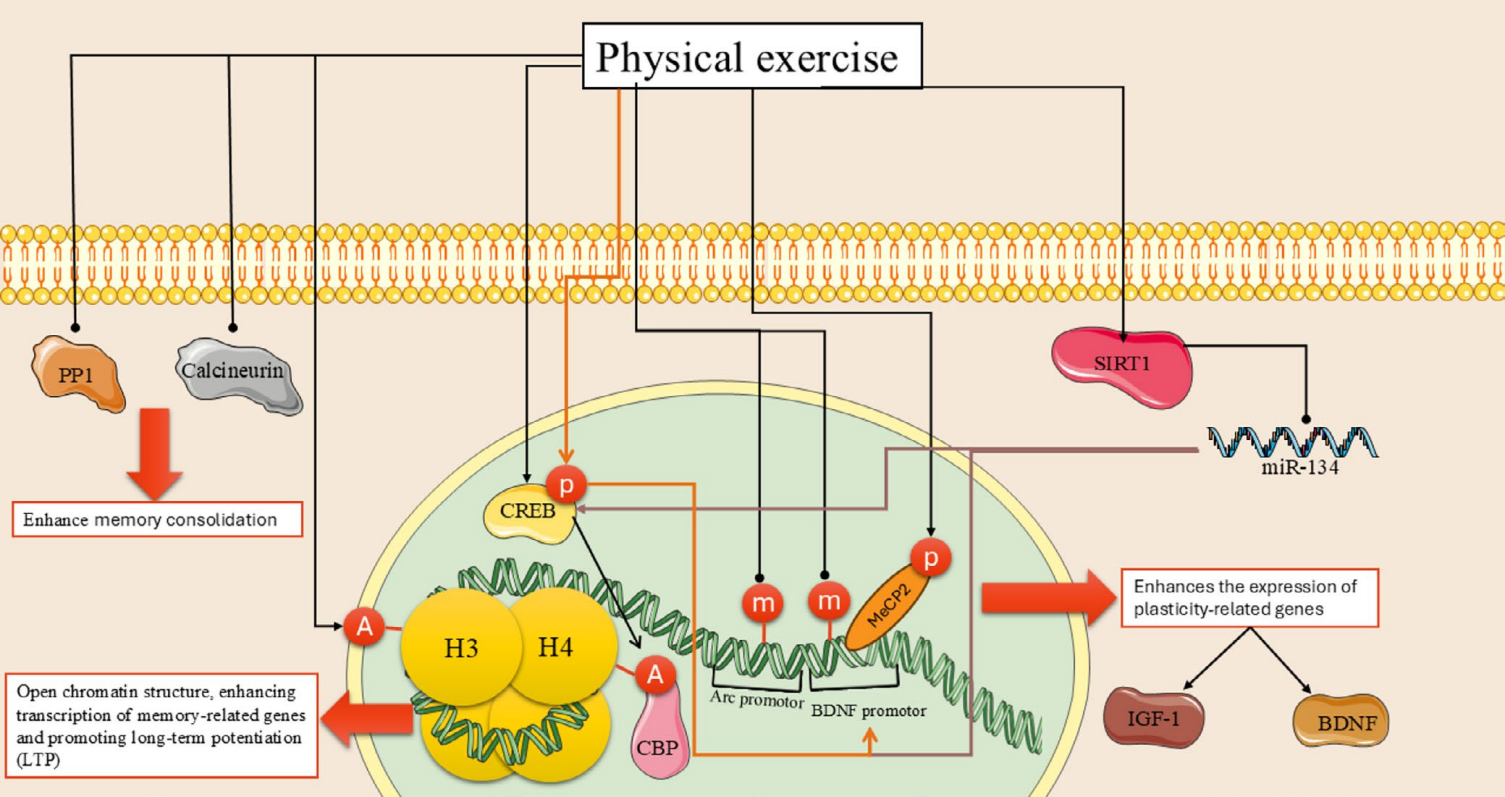
Epigenetic pathways linking exercise to enhanced BDNF expression and cognitive function. Physical activity appears to enhance cognitive performance through epigenetic regulation of brain‐derived neurotrophic factor (BDNF) expression. One proposed mechanism involves exercise‐induced glutamate release, which activates NMDA receptors (NMDA‐R) on postsynaptic neurons, triggering calcium (Ca^2+^) influx. The rise in intracellular Ca^2+^ levels initiates a cascade of intracellular signaling pathways, including CaMKII and MAPK/ERK/MSK, which are critical for transcriptional regulation. CaMKII‐mediated phosphorylation of methyl CpG‐binding protein 2 (MeCP2) leads to its dissociation from the *Bdnf* gene promoter, along with the release of associated transcriptional repressors such as Sin3A and histone deacetylases (HDACs). Additionally, exercise influences cellular metabolism in a way that upregulates SIRT1, a NAD^+^‐dependent deacetylase. SIRT1 may further contribute to MeCP2 release through deacetylation, suggesting a complementary SIRT1‐dependent epigenetic mechanism. Concurrently, CaMKII and MAPK/ERK/MSK signaling also promote the phosphorylation of specific histone proteins and activate the transcription factor CREB. This process facilitates the recruitment of CREB‐binding protein (CBP), a histone acetyltransferase (HAT), which modifies chromatin to adopt a more relaxed, transcriptionally active conformation. These molecular events collectively enable the transcription of the BDNF gene. Following translation, BDNF protein binds to its receptor TrkB, activating downstream signaling that supports synaptic plasticity and establishes a positive feedback loop. This feedback reinforces the neuroprotective and cognitive benefits associated with regular physical exercise.

Research shows exercise positively impacts learning and memory in humans and animals. In older adults, exercise improved cognition and countered age‐related decline, associated with hippocampal size changes. Children engaged in more aerobic exercise performed better on cognitive tests. Exercise acutely improved executive function and working memory in preadolescents and older adults. Studies found single bouts of aerobic and resistance exercise enhanced rat memory consolidation. Exercise is an effective therapy for depression, improving symptoms in randomized trials. It benefits brain diseases like Parkinson's, Alzheimer's, epilepsy, anxiety, traumatic brain injury (Babaei and Azari 2021; Chaire et al. 2020; Loprinzi et al. 2021). In rodents, memory improvements from exercise are associated with increased proliferation, neurogenesis, dendritic complexity, spine density, and molecular mechanisms involving neurotransmission, metabolism, plasticity. Exercise activates transcriptional machinery to epigenetically modulate learning/memory genes. BDNF and exercise can reduce depression and improve cognition through BDNF epigenetic regulation, building resistance. Hippocampal BDNF elevations improve learning in animals. Peripheral BDNF associates with cognition and human hippocampal size. Rodent studies link proteins upregulated by exercise to metabolism/plasticity through BDNF. The BDNF gene has nine exons regulated by methylation/histone modifications. Studies show exercise epigenetically regulates BDNF through acetylation/methylation, impacting plasticity/cognition. Exercise enhances BDNF and LTP via NMDA receptor activation. This activates signaling cascades involving CaMK, MAPK/ERK, CREB phosphorylation, which are important for BDNF‐mediated plasticity/cognition modulation by exercise. Blocking this signaling impairs exercise cognitive effects. CREB regulates gene transcription including BDNF by activating coactivators like CBP and HATs. CREB phosphorylation recruits CBP to initiate transcription. Studies show CBP acetylates histones and fails to compensate for the loss of CBP, indicating specificity. Exercise increases CBP and histone H3/H4 acetylation at BDNF promoters (Jemni et al. 2023; Phillips 2017). Acetylation promotes active chromatin for gene transcription, including activity‐regulated BDNF. Fear memory increases histone acetylation at BDNF Promoter IV. HDAC inhibitors increase BDNF and LTP/LTM. Histones undergo posttranslational modifications that interact, like H3S10 phosphorylation coupled to H3K14 acetylation. Fear memory activates MAPK/ERK via MSK1 contributing to H3 phosphorylation/acetylation (Figure 3). Learning increased H3 phosphoacetylation at BDNF Promoter IV. Environmental enrichment increased histone modifications and expression at BDNF in aged rats. Exercise reduced HDAC2 binding to increase BDNF. BDNF induces HDAC2 displacement, increasing histone acetylation at target genes like BDNF. MeCP2 silencing involves DNA methylation and binds methylated BDNF, recruiting repressors. Stimulation induces MeCP2 phosphorylation/dissociation from BDNF. Exercise reduced BDNF methylation and increased phospho‐MeCP2. Enrichment restored BDNF in Rett mice (Bredy et al. 2007; Palomer et al. 2016; Pathak et al. 2022). SIRT1 deacetylates MeCP2, preceding detachment from BDNF. SIRT1 loss impairs plasticity/memory via lowered BDNF/CREB and increased miR‐134. Exercise increases SIRT1 and attenuates its reduction in AD mice. SIRT1 cooperates with YY1 to repress miR‐134, facilitating BDNF transcription via CREB (Table 3) (Rasha et al. 2020; Wang et al. 2021; Zocchi and Sassone‐Corsi 2012).

**TABLE 3:** Molecular and epigenetic mechanisms by which exercise impacts cognition.

Mechanism	Effect on cognition	Molecular/epigenetic details	References
BDNF (Brain‐derived neurotrophic factor)	Enhances learning, memory, and mental health	Exercise increases BDNF levels, which are vital for hippocampal‐dependent spatial learning. BDNF expression is regulated by epigenetic modifications (e.g., acetylation, methylation) at its promoter regions. This is linked to improved cognitive function and resistance to neurological disorders	Gomez‐Pinilla et al. (2011)
Histone acetylation	Promotes memory formation and synaptic plasticity	Exercise increases acetylation of histones (H3, H4) at BDNF promoters, which opens chromatin, allowing transcription of BDNF and other genes associated with synaptic plasticity and memory. Acetylation at specific histone lysines (e.g., H4K8, H3) is linked to active transcription of cognitive‐related genes	Gomez‐Pinilla et al. (2011); Intlekofer et al. (2013)
CREB (cAMP response element‐binding protein)	Enhances long‐term memory (LTM) and synaptic plasticity	Exercise activates NMDA receptors, leading to Ca2+ influx and subsequent activation of CaMKII and MAPK/ERK pathways. This cascade phosphorylates CREB at serine 133, which is necessary for recruiting CBP (CREB‐binding protein) to initiate gene transcription, including BDNF. Blocking CaMKII or MAPK in the hippocampus reduces CREB activation and exercise‐induced cognitive improvements	Vaynman et al. (2003)
MeCP2 (Methyl CpG binding protein 2)	Regulates activity‐dependent gene expression	Exercise reduces MeCP2 binding to methylated DNA at BDNF promoter IV, promoting BDNF expression. MeCP2 phosphorylation (S421) is required to dissociate it from the promoter, allowing transcription of BDNF, which is crucial for synaptic plasticity and memory. In Mecp2 mutant mice (Rett syndrome model), exercise restores BDNF levels and improves cognitive deficits	Lonetti et al. (2010)
SIRT1 (Sirtuin 1)	Supports synaptic plasticity and memory formation	Exercise increases SIRT1 activity and expression in the hippocampus, which deacetylates MeCP2, leading to its dissociation from BDNF promoter IV and increased BDNF expression. SIRT1 also represses miR‐134, a microRNA that blocks CREB, facilitating BDNF transcription. SIRT1 loss of function impairs BDNF and CREB expression, leading to cognitive deficits	Gao et al. (2010)
Histone phosphorylation	Supports memory consolidation	Exercise induces phosphorylation of histone H3, which is coupled with acetylation (H3K14) in the hippocampus. This modification, particularly at BDNF promoter IV, is linked to transcriptional activation of BDNF during synaptic plasticity and memory formation	Chwang et al. (2007); Crosio et al. (2003)
DNA methylation	Modulates gene expression and memory	Exercise reduces DNA methylation at BDNF promoter IV, facilitating BDNF transcription. Demethylation of this region is crucial for allowing activity‐dependent BDNF expression, which is vital for synaptic plasticity and cognitive function	Gomez‐Pinilla et al. (2011)
miR‐134 (MicroRNA‐134)	Controls synaptic plasticity	Exercise decreases miR‐134 levels via SIRT1‐mediated repression. miR‐134 normally blocks CREB translation, so its reduction allows for increased CREB activity and BDNF transcription, facilitating synaptic plasticity and learning	Gao et al. (2010)

## 8 The Beneficial Effects of Polyphenols Combined With Exercise in Glioma or GBM

Polyphenols are abundant micronutrients in the diet, found in fruits, vegetables, and beverages, highlighting the importance of consuming a plant‐rich diet to obtain these beneficial compounds. These compounds are naturally occurring in plants and contribute to their color, flavor, and aroma. Polyphenols have garnered attention for their antioxidant and anti‐inflammatory properties, which can protect cells from damage and promote overall health. They can be classified into different groups according to their base structure, including flavonoids and stilbenes, each with unique properties and potential health benefits (Flores et al. 2016). Flavonoids are the most abundant group of polyphenols and are found in a variety of fruits, vegetables, and beverages. Stilbenes, such as resveratrol, are found in grapes, berries, and peanuts. Polyphenols exhibit low bioavailability due to interactions with the food matrix and metabolic processes, presenting a challenge for maximizing their health benefits (Di Lorenzo et al. 2021). Bioavailability refers to the extent to which a compound is absorbed into the bloodstream and reaches its target tissues. Factors such as the food matrix, gut microbiota, and metabolic enzymes can influence the bioavailability of polyphenols. Polyphenols exert neuroprotective actions by protecting neurons against injury induced by neurotoxins, reducing oxidative stress, and suppressing neuroinflammation (Vauzour 2012). Neurotoxins are substances that can damage or kill neurons, contributing to neurodegenerative diseases. Oxidative stress is an imbalance between the production of free radicals and the body's ability to neutralize them. Neuroinflammation is inflammation in the brain, which can damage neurons and impair cognitive function. They suppress neuroinflammation and promote memory and learning, contributing to cognitive enhancement and protection against age‐related decline (Vauzour 2012). By reducing neuroinflammation, polyphenols can protect neurons from damage and improve synaptic plasticity, the ability of synapses to strengthen or weaken over time. This, in turn, can enhance learning and memory. Beneficial effects involve decreases in oxidative/inflammatory stress signaling and increases in protective signaling, highlighting their role in promoting cellular resilience and maintaining brain homeostasis (Vauzour 2012).

Polyphenols can activate antioxidant enzymes, such as superoxide dismutase and catalase, which neutralize free radicals and protect cells from oxidative damage. They can also activate anti‐inflammatory pathways, reducing neuroinflammation and protecting neurons from damage. Resveratrol improves performance and prevents disease, amyloid‐beta toxicity, and hippocampal degeneration, making it a promising candidate for preventing and treating neurodegenerative diseases (Flores et al. 2016). Resveratrol is a stilbene found in grapes, berries, and peanuts. It has been shown to have antioxidant, anti‐inflammatory, and neuroprotective properties. Resveratrol can protect neurons from amyloid‐beta toxicity, a hallmark of Alzheimer's disease. Blueberry supplementation improves memory in older adults, suggesting that incorporating blueberries into the diet can enhance cognitive function and protect against age‐related decline (Krikorian et al. 2010). Blueberries are rich in anthocyanins, a type of flavonoid with antioxidant and anti‐inflammatory properties. Anthocyanins have been shown to improve memory, attention, and executive function. Flavonoids are associated with improved cognitive function across the lifespan, highlighting the importance of consuming a flavonoid‐rich diet to support brain health and cognitive performance (Barfoot et al. 2019). Flavonoids are a diverse group of polyphenols found in a variety of fruits, vegetables, and beverages. They have been shown to have antioxidant, anti‐inflammatory, and neuroprotective properties. Flavonoids can improve memory, attention, and executive function. Dietary intakes of curcumin have not been shown to prevent cognitive decline or improve cognitive performance. Despite its potential benefits, curcumin's effectiveness is limited by its poor bioavailability. Curcumin is a polyphenol found in turmeric with antioxidant and anti‐inflammatory properties, making it a promising candidate for promoting brain health and protecting against neurodegenerative diseases. Turmeric is a spice commonly used in Indian cuisine. Curcumin is the active ingredient in turmeric and is responsible for its yellow color. It has been shown to have a wide range of health benefits, including antioxidant, anti‐inflammatory, and anticancer properties. Poor bioavailability limits its effectiveness, requiring strategies to enhance absorption and delivery, such as combining it with piperine or formulating it into nanoparticles (Flores et al. 2016).

Bioavailability refers to the extent to which a compound is absorbed into the bloodstream and reaches its target tissues. Curcumin is poorly absorbed into the bloodstream and is rapidly metabolized, limiting its bioavailability. Piperine is a compound found in black pepper that can enhance the absorption of curcumin. Nanoparticles are tiny particles that can encapsulate curcumin and protect it from degradation, improving its bioavailability. Curcumin can modulate gene expression by influencing histone acetylation and DNA methylation, suggesting that it can alter the epigenetic landscape and influence neuronal function. Histone acetylation and DNA methylation are epigenetic modifications that regulate gene expression. Curcumin can inhibit histone deacetylases (HDACs), enzymes that remove acetyl groups from histones, leading to increased histone acetylation and gene activation. It can also inhibit DNA methyltransferases (DNMTs), enzymes that add methyl groups to DNA, leading to decreased DNA methylation and gene activation. It affects various pathways, including the sirtuin‐FoxO pathway and the Nrf‐2/ARE pathway, which are involved in cellular stress response, antioxidant defense, and longevity. The sirtuin‐FoxO pathway is a signaling pathway that regulates cellular stress response and longevity. Curcumin can activate sirtuins, enzymes that deacetylate proteins and regulate gene expression. The Nrf‐2/ARE pathway is a signaling pathway that regulates antioxidant defense. Curcumin can activate Nrf‐2, a transcription factor that binds to the antioxidant response element (ARE) and induces the expression of antioxidant genes. These processes maintain brain homeostasis and prevent neurodegenerative pathologies, highlighting the potential of curcumin as a protective agent against age‐related cognitive decline and neurodegenerative diseases (Vauzour 2012). By modulating gene expression and activating protective signaling pathways, curcumin can help maintain brain homeostasis and prevent neuronal damage. This can reduce the risk of age‐related cognitive decline and neurodegenerative diseases. Curcumin possesses antioxidant properties, reducing oxidative stress and accumulation, protecting neurons from damage, and promoting cognitive function [6]. Oxidative stress is an imbalance between the production of free radicals and the body's ability to neutralize them. Free radicals can damage cells and contribute to aging and disease. Curcumin can scavenge free radicals and protect cells from oxidative damage. It can reduce neuroinflammation and neuronal death by regulating inflammatory responses, potentially preventing the progression of neurodegenerative diseases (Jalouli et al. 2025). The investigation sought to assess the impact of endurance exercise and nanocurcumin supplementation on the short‐term memory capabilities of rats afflicted with GBM multiforme. A total of 35 healthy male Wistar rats were systematically allocated into seven distinct groups (*n* = 5 each): baseline healthy control, 4‐week healthy control, baseline cancer control, 4‐week cancer control, cancer + nanocurcumin, cancer + exercise, and cancer + exercise + nanocurcumin. Following a 1‐week period subsequent to the injection of tumor cells into the frontal cortex, the exercise intervention groups commenced a structured treadmill training program (18 m/min, 3 days per week for a duration of 4 weeks). The results indicated that the synergistic application of endurance exercise and nanocurcumin led to a significant reduction in tumor volume and an enhancement in short‐term memory in the cancer + exercise + nanocurcumin cohort when compared to the 4‐week cancer control group (Naghizadeh et al. 2023). Another investigation examined the implications of integrated resistance‐aerobic exercise and curcumin supplementation on the gene expression of Wnt/β‐catenin in the brain, alongside its relationship with the muscle‐derived myokine musclin within a GBM rat model. The exercise cohorts participated in a regimen of combined aerobic and resistance training three times weekly over a duration of 4 weeks. Nanocurcumin was administered via oral route at a dosage of 80 mg/kg, 5 days each week for a period of 4 weeks. All tumor‐bearing cohorts demonstrated a significantly heightened expression of Wnt/β‐catenin gene when contrasted with healthy control subjects. Nevertheless, the concurrent application of exercise and nanocurcumin yielded the most substantial downregulation of Wnt/β‐catenin expression. Importantly, a negative correlation between the mRNA levels of Wnt and musclin was identified within the tumor group treated with nanocurcumin (Afsharyousefi et al. 2024).

An investigation sought to evaluate the impact of endurance training and nanocurcumin supplementation on the expression of miR‐21 and P53 genes within brain tumor tissue utilizing a GBM animal model. A total of 35 male Wistar rats, aged 8 weeks, were systematically allocated into seven distinct groups: healthy control, 4‐week healthy control, initial cancer control, 4‐week cancer control, cancer + training, cancer + nanocurcumin, and cancer + combined training + nanocurcumin. One week following the injection, the subjects commenced a 4‐week treadmill endurance training regimen, while nanocurcumin was administered orally at a dosage of 80 mg/kg on a daily basis for a duration of 28 days. The findings indicated that the expression levels of miR‐21 were markedly diminished in the training, nanocurcumin, and combined intervention groups when compared to the 4‐week cancer control. Furthermore, P53 expression was significantly elevated in both the nanocurcumin and combined training + nanocurcumin groups in comparison with the cancer and 4‐week cancer controls, thereby suggesting an augmentation of tumor‐suppressive activity (Naghizadeh et al. 2024). In an independent investigation, scholars examined the synergistic effects of moderate‐intensity continuous physical activity and nanocurcumin supplementation on the modulation of the STAT3 gene expression within a murine model of GBM multiforme. The results indicated that the cohort receiving both interventions (exercise in conjunction with nanocurcumin) exhibited a marked decrease in tumor volume and STAT3 gene expression. Conversely, this decrement was not observed to reach statistical significance in the groups that were administered either exercise alone or nanocurcumin alone (Hajinajaf et al. 2022). Neuroinflammation is inflammation in the brain, which can damage neurons and impair cognitive function. Curcumin can inhibit the production of proinflammatory cytokines, reducing neuroinflammation and protecting neurons from damage. Curcumin also shows promise in modulating cellular signaling pathways associated with viability and cognitive function, suggesting that it can influence neuronal survival and synaptic plasticity (Jalouli et al. 2025). Cellular signaling pathways are networks of proteins that communicate with each other to regulate cellular function. Curcumin can modulate cellular signaling pathways involved in neuronal survival, growth, and differentiation. It can also enhance synaptic plasticity, the ability of synapses to strengthen or weaken over time. Quercetin is a naturally nontoxic flavonoid with antioxidant, anti‐apoptotic, and anti‐inflammatory properties, making it a promising candidate for promoting brain health and protecting against neurodegenerative diseases. Flavonoids are a diverse group of polyphenols found in a variety of fruits, vegetables, and beverages. Quercetin is one of the most abundant flavonoids in the diet. It is found in various plants and exhibits potential therapeutic applications, including the prevention and treatment of age‐related diseases and neurodegenerative conditions. Quercetin is found in a variety of plants, including onions, apples, berries, and broccoli. It has been shown to have a wide range of health benefits, including antioxidant, anti‐inflammatory, and anticancer properties (Cui et al. 2022). Quercetin can modulate mitochondrial functions and scavenge free radicals, protecting cells from oxidative damage and promoting cellular energy production (Gibellini et al. 2015).

Mitochondria are organelles responsible for producing energy in cells. Quercetin can protect mitochondria from damage and improve their function. It can also scavenge free radicals and protect cells from oxidative damage, playing a crucial role in regulating cellular stress response, metabolism, and longevity (Cui et al. 2022). Sirtuins are a family of enzymes that deacetylate proteins, removing acetyl groups from them. Deacetylation can alter protein function and regulate gene expression. SIRT1 is the most well‐studied sirtuin and has been shown to have a wide range of health benefits. Quercetin regulates cellular senescence and multiple processes, including oxidative stress and inflammatory response via SIRT1, highlighting its ability to modulate key pathways involved in aging and disease. Cellular senescence is a process in which cells stop dividing and enter a state of irreversible growth arrest. Senescent cells can accumulate with age and contribute to tissue dysfunction and disease. Quercetin can inhibit cellular senescence and protect against age‐related diseases. It also controls phosphorylation, mitochondrial damage, and autophagy through SIRT1‐related pathways, suggesting that it can influence a variety of cellular processes critical for neuronal survival and cognitive function. Phosphorylation is the addition of a phosphate group to a protein, which can alter its function. Mitochondrial damage can lead to cellular dysfunction and death. Autophagy is a process in which cells degrade and recycle damaged or unnecessary components. Quercetin can protect against mitochondrial damage and promote autophagy (Cui et al. 2022). Quercetin has beneficial effects against diseases such as Alzheimer's, Parkinson's, and Huntington's, suggesting that it can protect neurons from damage and improve cognitive function in these conditions. Alzheimer's disease is a neurodegenerative disease characterized by memory loss and cognitive decline. Parkinson's disease is a neurodegenerative disease characterized by motor dysfunction. Huntington's disease is a neurodegenerative disease characterized by cognitive and motor dysfunction (Cui et al. 2022). It also shows promise in treating depression, osteoporosis, and myocardial ischemia, highlighting its potential as a multitarget therapeutic agent. Depression is a mood disorder characterized by sadness, loss of interest, and fatigue. Osteoporosis is a condition characterized by weak and brittle bones. Myocardial ischemia is a condition in which the heart muscle does not receive enough blood flow (Cui et al. 2022). Quercetin's neuroprotective effects are linked to the SIRT1/FoxG1/CREB/BDNF/Trk‐catenin pathway, indicating that it can influence key signaling molecules involved in neuronal survival, growth, and differentiation. FoxG1 is a transcription factor that regulates brain development. CREB is a transcription factor that regulates learning and memory. BDNF is a neurotrophin that promotes neuronal survival, growth, and differentiation. Trk‐catenin is a signaling pathway that regulates cell adhesion and migration (Cui et al. 2022).

Epigallocatechin‐3‐gallate (EGCG) is found in green tea and other plant sources, making green tea a valuable dietary source of this beneficial polyphenol. Green tea is made from the leaves of the *Camellia sinensis* plant. It is rich in polyphenols, including EGCG. It is a polyphenol with antioxidant and anti‐inflammatory properties, contributing to its potential health benefits. Antioxidants protect cells from damage caused by free radicals. Anti‐inflammatory agents reduce inflammation, which can damage cells and contribute to disease (Flores et al. 2016). EGCG can modulate gene expression and cellular signaling pathways, influencing a variety of cellular processes. Gene expression is the process by which cells read and translate genes into proteins. Cellular signaling pathways are networks of proteins that communicate with each other to regulate cellular function (Flores et al. 2016). EGCG possesses antioxidant properties, reducing oxidative stress and protecting cells from damage, which is crucial for maintaining neuronal health and cognitive function. Oxidative stress is an imbalance between the production of free radicals and the body's ability to neutralize them. Free radicals can damage cells and contribute to aging and disease. It inhibits NFKB and cyclooxygenase‐2 (COX‐2), reducing inflammation, which can protect neurons from damage and improve cognitive function. NFKB is a transcription factor that regulates the expression of proinflammatory genes. COX‐2 is an enzyme that produces proinflammatory molecules (Flores et al. 2016). EGCG induces apoptosis in cancer cells and modulates immune system response, highlighting its potential as an anticancer agent (Niedzwiecki et al. 2016). Apoptosis is programmed cell death. It is a normal process that helps to remove damaged or unnecessary cells. EGCG demonstrates neuroprotective effects and can improve synaptic plasticity, suggesting that it can protect neurons from damage and enhance cognitive function. Synaptic plasticity is the ability of synapses to strengthen or weaken over time in response to experience. It is essential for learning and memory. It may enhance cognitive function and prevent age‐related cognitive decline, making it a promising candidate for promoting brain health throughout life (Coppedè 2022). Age‐related cognitive decline is a gradual decline in cognitive function that occurs with age. It can affect memory, attention, and executive function. Further research is needed to fully understand its role and establish it as an advanced target for neuroinflammatory conditions, emphasizing the need for continued investigation into its therapeutic potential. Neuroinflammation is inflammation in the brain, which can damage neurons and impair cognitive function. Blueberries contain polyphenolic compounds, mainly anthocyanins, with antioxidant and anti‐inflammatory effects, contributing to their potential benefits for brain health (Krikorian et al. 2010). Anthocyanins are a type of flavonoid that gives blueberries their blue color. They have antioxidant and anti‐inflammatory properties. Green tea contains epigallocatechin‐3‐gallate (EGCG) and other polyphenols with neuroprotective properties, making it a valuable dietary component for promoting brain health (Niedzwiecki et al. 2016). EGCG is a polyphenol that has antioxidant, anti‐inflammatory, and neuroprotective properties. Both blueberries and green tea are rich in antioxidants and other bioactive compounds that can benefit brain function, highlighting the importance of incorporating these foods into a brain‐healthy diet (Neto 2007). Antioxidants protect cells from damage caused by free radicals. Bioactive compounds are substances that have a biological effect on the body. Combining blueberries and green tea may result in synergistic effects due to their complementary mechanisms of action, potentially leading to enhanced antioxidant activity and neuroprotection. Synergistic effects occur when the combined effect of two or more substances is greater than the sum of their individual effects. The combination can enhance antioxidant activity, reduce inflammation, and promote neuronal signaling, contributing to improved cognitive function and brain health. Neuronal signaling is the communication between neurons. It is essential for brain function. Synergistic effects may improve cognitive function and protect against neurodegenerative diseases more effectively than individual components, suggesting that combining these foods may offer greater benefits than consuming them separately (Jalouli et al. 2025).

Neurodegenerative diseases are diseases that cause the progressive loss of neurons. Blueberry supplementation improves memory in older adults, suggesting that incorporating blueberries into the diet can enhance cognitive function and protect against age‐related decline (Krikorian et al. 2010). Older adults are at increased risk of cognitive decline. Blueberries have been shown to improve memory, attention, and executive function in older adults. Green tea extract enhances cognitive function and improves synaptic plasticity, highlighting its potential as a cognitive enhancer (Coppedè 2022). Synaptic plasticity is the ability of synapses to strengthen or weaken over time in response to experience. It is essential for learning and memory. Combined interventions may offer enhanced cognitive and cardiovascular benefits, suggesting that combining blueberries and green tea may offer greater benefits than consuming them separately (Cheng et al. 2024). Cardiovascular benefits include improved blood pressure, cholesterol levels, and blood vessel function. Berberine is a natural compound found in several plants, highlighting its availability in various traditional medicine systems. Berberine is found in plants such as goldenseal, barberry, and Oregon grape. It has been used in traditional medicine for centuries. It exhibits various biological activities, including antioxidant, anti‐inflammatory, and neuroprotective effects, contributing to its potential benefits for brain health. Antioxidants protect cells from damage caused by free radicals. Anti‐inflammatory agents reduce inflammation, which can damage cells and contribute to disease (Flores et al. 2016).

**FIGURE 4:**
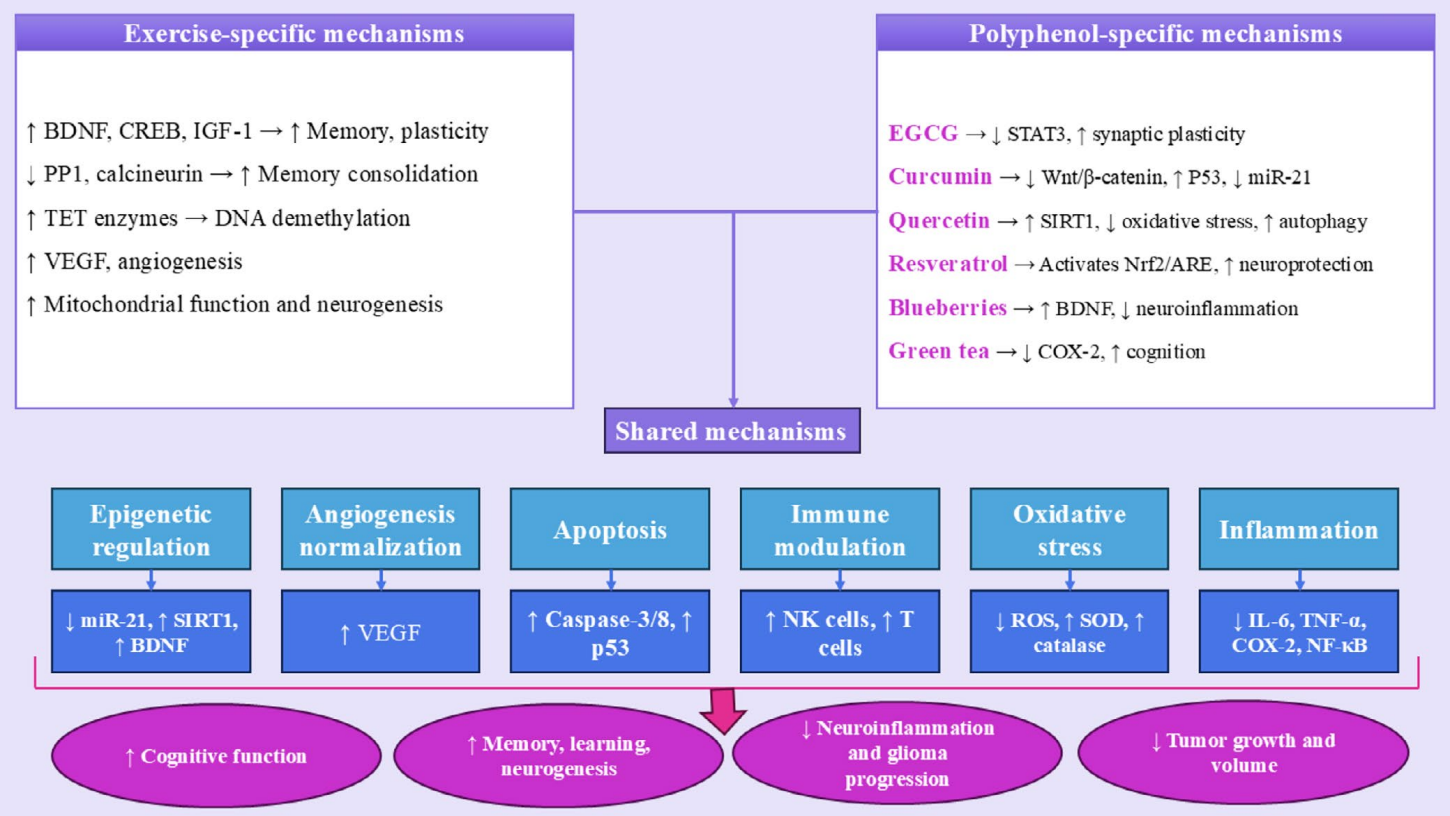
Synergistic effects of polyphenol intake and physical exercise on glioma and glioblastoma outcomes.

Berberine's bioavailability is limited, but strategies to enhance absorption are being explored, such as using nanoparticles or combining it with other compounds (Flores et al. 2016). Bioavailability refers to the extent to which a compound is absorbed into the bloodstream and reaches its target tissues. Berberine is poorly absorbed into the bloodstream and is rapidly metabolized, limiting its bioavailability. Soy contains isoflavones, which are polyphenolic compounds with estrogen‐like activity, making soy a unique dietary source of these compounds. Isoflavones are a type of flavonoid that is found in soy. They have estrogen‐like activity, meaning that they can bind to estrogen receptors and exert estrogen‐like effects in the body. Soy isoflavones may have cognitive benefits, particularly in postmenopausal women, suggesting that they can help protect against age‐related cognitive decline. Postmenopausal women are at increased risk of cognitive decline due to the decline in estrogen levels. Soy isoflavones have been shown to improve memory, attention, and executive function in postmenopausal women. They can modulate hormonal pathways and influence brain function, highlighting the complex interplay between hormones and cognition (Durazzo et al. 2019). Hormones play a crucial role in regulating brain function. Soy isoflavones can modulate hormonal pathways and influence brain function. Combining berberine and soy isoflavones with exercise may offer synergistic benefits for brain health, potentially leading to enhanced cognitive function and protection against neurodegenerative diseases. Synergistic effects occur when the combined effect of two or more substances is greater than the sum of their individual effects. This approach could target multiple pathways, including antioxidant defense, inflammation, and hormonal regulation, providing a comprehensive strategy for promoting brain health. Antioxidant defense protects cells from damage caused by free radicals. Inflammation can damage cells and contribute to disease. Hormonal regulation is the process by which hormones are regulated in the body. Further research is needed to explore the potential of these combined interventions in preventing and treating neurodegenerative diseases, emphasizing the need for continued investigation into their therapeutic potential (Di Liegro et al. 2019; Jalouli et al. 2025). Neurodegenerative diseases are diseases that cause the progressive loss of neurons. An investigation was undertaken to evaluate the impact of aquatic exercise and a nanoliposome‐encapsulated supplement comprising four distinct herbal extracts within a rat model of midbrain neoplasm. In this empirical study, 56 male Wistar rats were systematically allocated into eight distinct cohorts: normal control, tumor model, model + exercise, model + nanoliposome, model + crude extract, model + nanoliposome‐extract, model + extract + exercise, and model + nanoliposome‐extract + exercise. The midbrain tumor was elicited through stereotaxic administration of C6 glioma cells (5 × 10^5^ cells) into the substantia nigra region. Over a duration of 6 weeks, the subjects were administered either the nanoformulated herbal extract (100 mg/kg/day), crude extract (100 mg/kg/day), and/or engaged in swimming training. The results indicated that the synergistic application of aquatic exercise and the nanoliposome‐enhanced herbal formulation markedly elevated the expression levels of P53 and Hras, while concurrently diminishing the expression of IL‐10 and Casp8, thereby implying potential antitumor and immunomodulatory properties (Farajizadeh et al. 2023) (Figure 4).

## 9 Upcoming Directions of Exercise‐Based Interventions in Glioma Therapy and Maintaining Brain's Normal Functions

The integration of exercise into glioma therapy is gaining traction as a promising adjunctive treatment aimed at improving patient outcomes. Current research indicates that exercise can alleviate symptoms associated with glioma, enhance cognitive function, and potentially influence tumor biology through epigenetic mechanisms. As this field evolves, several future directions can be considered to optimize the therapeutic benefits of exercise for glioma patients. Future studies should focus on developing personalized exercise regimens tailored to individual patient needs, considering factors such as tumor grade, treatment stage, and overall health status. A recent initiative, the ActiNO program, has shown that individualized exercise plans can be both feasible and safe for glioma patients, allowing for adaptations based on symptoms and physical capabilities. This approach could enhance adherence and maximize the benefits of exercise (Jost et al. 2023).

While preliminary studies have demonstrated positive outcomes from exercise interventions, larger randomized controlled trials (RCTs) are essential to validate these findings. For instance, a pilot study indicated improvements in cognitive function among glioma patients participating in a structured exercise program. Expanding these trials to include diverse populations and longer follow‐up periods will provide more robust data on the efficacy of exercise in this patient group (Gehring et al. 2020). Research should delve deeper into the mechanistic pathways through which exercise impacts glioma biology and cognitive function. Understanding how exercise induces epigenetic changes that may affect tumor progression and cognitive health could lead to more targeted interventions. This includes studying the role of exercise‐induced factors like brain‐derived neurotrophic factor (BDNF) and their influence on neuroplasticity and tumor suppression. Combining exercise with other therapeutic modalities may enhance overall treatment effectiveness. Integrating physical activity with cognitive training or nutritional support could address multiple aspects of glioma management. For example, concurrent interventions that focus on both physical fitness and cognitive rehabilitation may lead to synergistic effects, improving both physical and mental health outcomes.

Identifying and addressing barriers that prevent glioma patients from engaging in regular physical activity is crucial. Factors such as fatigue, mobility issues, and lack of motivation can hinder participation in exercise programs. Future research should explore strategies to overcome these barriers, such as providing remote coaching or incorporating community support systems. Longitudinal studies assessing the long‐term impacts of exercise on quality of life in glioma patients are needed. These studies should evaluate not only physical health but also psychological well‐being, social functioning, and overall life satisfaction over time. Understanding how sustained physical activity influences quality of life can inform clinical practice and patient support strategies. In conclusion, the future directions of exercise in glioma therapy hold significant promise for enhancing patient care. By focusing on personalized approaches, conducting larger trials, exploring underlying mechanisms, integrating multimodal therapies, addressing barriers to participation, and evaluating long‐term quality‐of‐life outcomes, healthcare providers can better harness the benefits of exercise as a vital component of glioma management (Keats et al. 2022; Nowak et al. 2023).

## 10 Conclusions and Key Messages

The integration of polyphenol‐based nutritional strategies with structured exercise interventions presents a promising and holistic approach to enhancing brain function and controlling glioma progression. These interventions synergistically modulate key molecular and epigenetic mechanisms, including DNA methylation, histone modifications, and noncoding RNAs, which are central to both cognitive processes and tumor biology. By targeting neuroinflammation, oxidative stress, neurogenesis, and cellular plasticity, this combination offers a powerful dual‐targeting strategy that benefits both neural integrity and tumor suppression. Importantly, this approach aligns with the principles of predictive, preventive, and personalized medicine, offering low‐cost, noninvasive strategies that can be tailored to individual patient needs. The evidence indicates that such interventions can enhance learning, memory, and overall cognitive resilience while contributing to the regulation of oncogenic pathways in glioma. From a practical standpoint, the accessibility and safety of polyphenols and exercise make them viable components of adjunct therapy in both preclinical and clinical contexts. Despite increasing interest in exercise and dietary polyphenols separately, few studies have explored their combined impact on brain function and glioma with a specific focus on epigenetic regulation and cognitive performance. This review fills a critical gap in the literature by synthesizing evidence from neuroscience, oncology, and molecular epigenetics to provide a comprehensive perspective. Future research should focus on translational and clinical studies that assess the integrative effects of these interventions, using molecular biomarkers to evaluate therapeutic efficacy. This combined strategy holds great promise for improving brain health, delaying glioma progression, and enhancing quality of life in affected individuals.

## 11 Future Perspectives

Future research should prioritize well‐designed clinical trials to evaluate the synergistic effects of specific polyphenols—such as resveratrol, curcumin, and EGCG—combined with defined exercise regimens (e.g., aerobic, resistance, or combined protocols) on cognitive outcomes and glioma progression. These studies should integrate multiomics approaches, including epigenomic profiling, to identify precise molecular targets and biomarkers of response. Additionally, investigations into the optimal dose, timing, and bioavailability of polyphenols in conjunction with exercise are essential for clinical translation. Personalized protocols based on individual genetic and epigenetic profiles will enhance therapeutic efficacy and safety. Advancing this integrated approach may lead to evidence‐based, nonpharmacological interventions that support brain function and inhibit glioma development with minimal side effects.

## Author Contributions

**Guobiao Yang:** conceptualization (equal), data curation (equal), investigation (equal), methodology (equal), validation (equal), visualization (equal), writing – original draft (equal), writing – review and editing (equal), supervision (equal with Farzam Kiarasi). **Wanying Yang:** conceptualization (equal), data curation (equal), investigation (equal), methodology (equal), validation (equal), visualization (equal), writing – original draft (equal), writing – review and editing (equal). **Farzam Kiarasi:** conceptualization (equal), data curation (equal), investigation (equal), methodology (equal), supervision (equal), validation (equal), visualization (equal), writing – original draft (equal), writing – review and editing (equal).

## Consent

The authors have nothing to report.

## Conflicts of Interest

The authors declare no conflicts of interest.

## Data Availability

Data sharing not applicable to this article as no datasets were generated or analyzed during the current study.
